# Leaf wound induced ultraweak photon emission is suppressed under anoxic stress: Observations of *Spathiphyllum* under aerobic and anaerobic conditions using novel *in vivo* methodology

**DOI:** 10.1371/journal.pone.0198962

**Published:** 2018-06-14

**Authors:** Carl L. Oros, Fabio Alves

**Affiliations:** 1 Information Sciences Department, Graduate School of Operational and Information Sciences, Naval Postgraduate School, Monterey, California, United States of America; 2 Physics Department, Graduate School of Engineering and Applied Sciences, Naval Postgraduate School, Monterey, California, United States of America; Massachusetts General Hospital, UNITED STATES

## Abstract

Plants have evolved a variety of means to energetically sense and respond to abiotic and biotic environmental stress. Two typical photochemical signaling responses involve the emission of volatile organic compounds and light. The emission of certain leaf wound volatiles and light are mutually dependent upon oxygen which is subsequently required for the wound-induced lipoxygenase reactions that trigger the formation of fatty acids and hydroperoxides; ultimately leading to photon emission by chlorophyll molecules. A low noise photomultiplier with sensitivity in the visible spectrum (300–720 nm) is used to continuously measure long duration ultraweak photon emission of dark-adapting whole *Spathiphyllum* leaves (*in vivo*). Leaves were mechanically wounded after two hours of dark adaptation in aerobic and anaerobic conditions. It was found that (1) nitrogen incubation did not affect the pre-wound basal photocounts; (2) wound induced leaf biophoton emission was significantly suppressed when under anoxic stress; and (3) the aerobic wound induced emission spectra observed was > 650 nm, implicating chlorophyll as the likely emitter. Limitations of the PMT photocathode’s radiant sensitivity, however, prevented accurate analysis from 700–720 nm. Further examination of leaf wounding profile photon counts revealed that the pre-wounding basal state (aerobic and anoxic), the anoxic wounding state, and the post-wounding aerobic state statistics all approximate a Poisson distribution. It is additionally observed that aerobic wounding induces two distinct exponential decay events. These observations contribute to the body of plant wound-induced luminescence research and provide a novel methodology to measure this phenomenon *in vivo*.

## Introduction

### Plant photo-chemical wounding responses

Plants have evolved a variety of means to energetically sense and respond to abiotic and biotic environmental stress. Two typical photochemical signaling responses involve the emission of volatile organic compounds (VOC) and light. Plants are known to release VOCs and green leaf volatiles (GLVs) when tissues are injured [1–4]. Both of these emission types are known to occur naturally (i.e. they are self-induced) [5] or they can be induced in response to abiotic stress (i.e., temperature, wind, wounding) as well as damage inflicted by herbivores, fungicides, viruses, and bactericides [3]. GLVs are derived from the peroxidation of polyunsaturated fatty acids (PUFAs) and are considered the major VOC constituents released by plants. These volatiles are believed to play a role in plant signaling and herbivore plant defense [3,6,7].

In addition to chemical emissions, all living organisms emit a low intensity luminescence which is spontaneous (or self-induced) as well as induced via injury [8–14]. This phenomenon has been extensively studied in numerous plants, animals, and humans. Further, diseased and injured cells have also been observed to emit more light than healthy ones. This low-level photoemission has been referred to as chemiluminescence (CL), biophotons (Popp), biophoton emission, ultraweak photon emission (UPE), ultraweak bioluminescence, autoluminescence, and self-bioluminescent emission (SBE). (We use the terms UPE, biophoton, and photoemission interchangeably throughout this paper). Research has revealed that light emitted due to the plant wounding process is related to chlorophyll [15–17], namely singlet chlorophyll (^1^Chl*) within the injured chloroplasts [13,18] and that oxygen and lipoxygenase (LOX) are two of the key initiating chemical components essential for wound induced volatiles and biophoton emission.

Lipoxygenase is central to both wound induced volatile chemical and photon emission and requires oxygen for its catalyzing reaction [12,19]. Proton-transfer-reaction mass spectrometry (PTR-MS) analysis of beech and aspen leaf wound induced VOCs in both oxygen (O_2_) and nitrogen (N_2_) atmospheres revealed that the formation of the hexyl and hexenyl family of compounds was dependent upon oxygen which is essential for the lipoxygenase (LOX) reaction to trigger the formation of fatty acids and hydroperoxides [1]. Fall et al. also discovered that the emission of the hexenyl family of compounds was not dependent upon light and that there was no pooling of C_6_ aldehydes or alcohols in the unwounded leaves. Similar experiments with *Phragmites australis* reported that the emission of all VOCs, with the exception of acetaldehyde, were oxygen dependent and undetectable in an N_2_ atmosphere [2].

It has been shown that lipid peroxidation is the main source of stress induced photo emission in plants [13,15] and that lipoxygenase (LOX2) is responsible for the formation of GLVs in Arabidopsis leaves [20]. Lipoxygenases are enzymes that catalyze oxygenation of polyunsaturated fatty acids to form fatty acid hydroperoxides (HPO). Recent studies employing Singlet Oxygen Sensor Green (SOSG) florescence imaging [18,21] have shown that singlet oxygen is produced in response to leaf wounding even in the dark. Employment of lipoxygenase inhibitors has also been shown to restrict the formation of triplet carbonyls (^3^L = O*) that ultimately prevent the formation of singlet oxygen (^1^O_2_) in wounded Arabidopsis leaves and bacteria [16,18,22]. These results mutually support the observation that disruption of the lipid peroxidation pathway by inhibiting lipoxygenase—either through elimination of O_2_ or through the use of inhibitors (i.e. catechol)—prevents both the hexenyl family VOC emission and ^1^O_2_ production. Current research suggests that singlet oxygen and singlet chlorophyll within damaged chloroplasts are mutually implicated in the wound induced photo luminescence process, leading to chlorophylls as the final photon emitters [13].

We contribute to this body of research by showing that wound-induced leaf photon emission of whole *Spathiphyllum* leaves (*in vivo*) is inhibited when under anoxic stress. Spectral analysis using optical edgepass filters reveal the preponderance of photoemission is > 650 nm. This supports published research identifying chlorophyll molecules as the main emitters of wound induced UPE. These findings support experimental data provided in [18] that inhibition of lipoxygenase production directly effects singlet oxygen production; ultimately inhibiting singlet chlorophyll luminescence.

#### Plant anaerobic stress

Plants depend on a continuous supply of environmental oxygen to support life-sustaining biological processes. The effects of plant tissue hypoxia, or anoxia on UPE (or chemiluminescence, as it was termed) has been known and studied for some time [12,23–26]. Italian and Russian researchers were the initial groups to report on UPE dependence on oxygen in whole plants and extracts of non-chlorophyll containing (etiolated) seedlings of wheat, beans, lentils, and corn [27] and wheat roots [28]. Research involving a variety of plant types: barley seedlings [29], spinach, hibiscus [30], peas, beans, corn seedlings and roots [31] and pumpkin roots and seedlings [32] in different types of anoxic gasses (N_2_, CO_2_, Ar) have shown that luminescence decreases significantly when plants are placed under anaerobic stress (~90% in N_2_ [31]). Further, UPE due to peroxidation of membrane lipids was previously found to be detectable only when oxygen was present in the medium [32]. Many of these early studies were performed with first generation photomultiplier tubes (PMTs) and liquid scintillation spectrophotometers [31] with detached / destroyed leaves or leaf and biochemical extracts. Not many UPE experiments, including more recent ones, have examined whole plants *in vivo*. Our results extend previous research and show that that short periods of anaerobic stress do not completely extinguish UPE and wound induced UPE is significantly inhibited under anoxic conditions. We additionally provide a novel methodology to examine wound induced UPE in aerobic and anaerobic environments.

## Results and discussion

### Spontaneous ultra-weak photon emission from *Spathiphyllum* leaves

**Fig 1:**
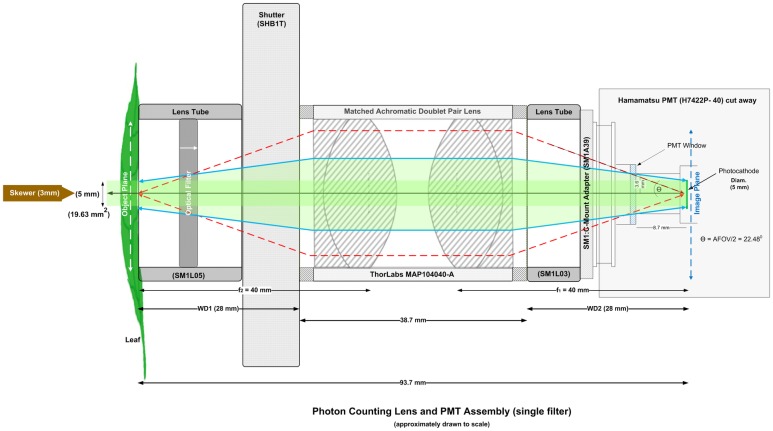
Primary photon counting lens and PMT assembly. Primary PMT lens configuration with matched achromatic doublet pair lens (*f* = 40 mm) and mechanical shutter (SHB1T). Single edgepass filters were manually inserted as indicated when used for spectroscopy measurements.

Spontaneous ultra-weak photon emission from *Spathiphyllum* leaves was measured *in vivo* with a low noise, high quantum efficiency (QE; 580 nm) electronically cooled PMT model H7422P-40 (Hamamatsu Photonics, K.K., Iwata City, Japan) fitted with a matched achromatic doublet pair lens (see Fig 1).

This matched lens pair forms an image relay system where the image height is equal to the photocathode diameter. (See Material and methods section for more details). The spectral response of the PMT is in the visible wavelength band (300–720 nm). All experiments were carried out in a controlled, nested dark room lab environment (see Materials and methods section). Whole plants were placed in a dark box enclosure while under non-actinic green LED light. The selected upper leaf epidermis was placed facing the photocathode in the object plane. Care was taken to ensure leaves were not unintentionally wounded during emplacement. Once secured in place, the plants were allowed to dark-adapt for two hours prior to mechanical wounding. Plants were wounded by manually piercing the lamina between the midrib and veins with a 3 mm diameter bamboo skewer approximately centered in the object plane. Continuous photon counting was performed from fluorescent decay throughout the wounding-decay sequence. Dark count measurements were crosschecked against historical data by closing the lens shutter at the completion of each experiment. Dark counts never exceeded 5 cps. Two non-blooming Spaths (of non-petite variety) were used for the wounding and single filter spectroscopy measurements. Only healthy, unwounded leaves were used for these tests. The spectral filter wheel test was performed with an early blooming spath on a previously wounded leaf.

### Biophoton decay analyses

#### Pre-wounding fluorescent decay (dark adaptation)

**Fig 2:**
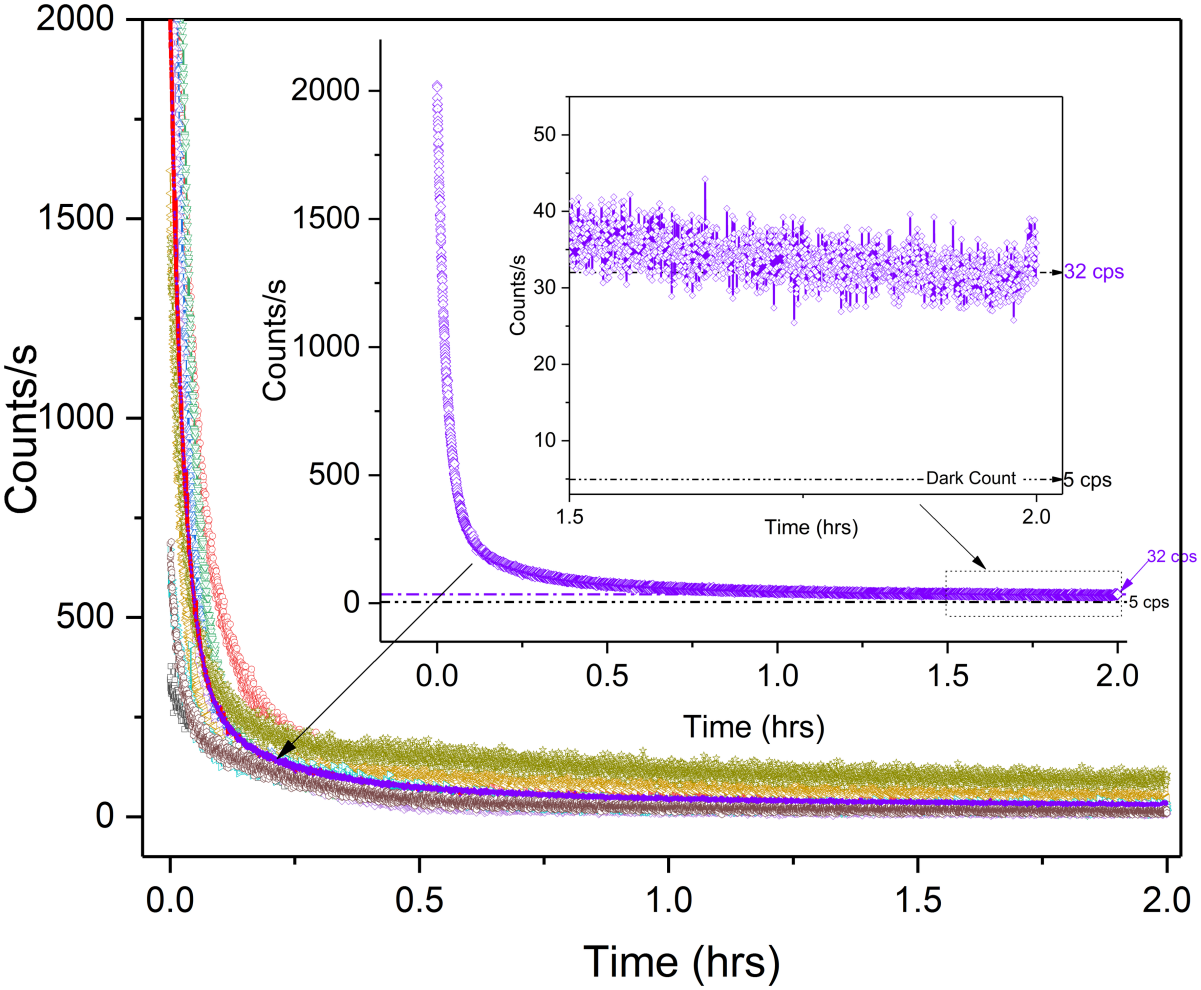
*Spathiphyllum* dark-adaptation decay plots. Nine sample decay plots of dark-adapting *Spathiphyllum*. Purple curve (and inset) depicts the sample average. Enlarged scale of basal count (1.5–2.0 hrs.) reveals 32 cps after two hours. Average dark count is 5 cps.

Fig 2 shows the two-hour fluorescent decay plots recorded in the visual wavelength band (300–720 nm) for nine different dark-adapting *Spathiphyllum* leaves previously exposed to routine, overhead laboratory white LED lighting. We chose a two hour pre-wounding dark adaptation time to be consistent with similar studies [15,17]. The nine individual decay plots were averaged (Fig 2). Only extreme outliers (> 2.7 σ) attributable to PMT shot noise or other electrical disturbances were masked and removed from data calculations. It should be noted that the overall fluorescent decay required ~2 hours for the decay rate to minimize. However, the photoemission never reached zero and yielding an average basal photocount of 32 cps (27 cps greater than the typical dark count of the PMT). Our data clearly shows a significant basal photo luminescence well after what is considered normal chlorophyll fluorescent decay.

The average decay data was then fit to both double exponential and hyperbolic decay functions. The double exponential decay curve was fit to the equation:

$$y=A_{1}e^{\left(-x/t_{1}\right)}+A_{2}e^{\left(-x/t_{2}\right)}+y_{0}$$ (1)

where (y_0_ = 36.5; A_1_ = 1721; t_1_ = 99; A_2_ = 245; and t_2_ = 955).

The hyperbolic decay curve was fit to the equation:

$$f=ax^{2}+bxy+cy^{2}+dx+e(y-1)$$ (2)

where (a = 2.1 x 10^-8^, b = 1.2 x 10^-5^, c = 3.4 x 10^-7^, d = -3.8 x 10^-4^, e = -2.7 x 10^-4^).

**Fig 3:**
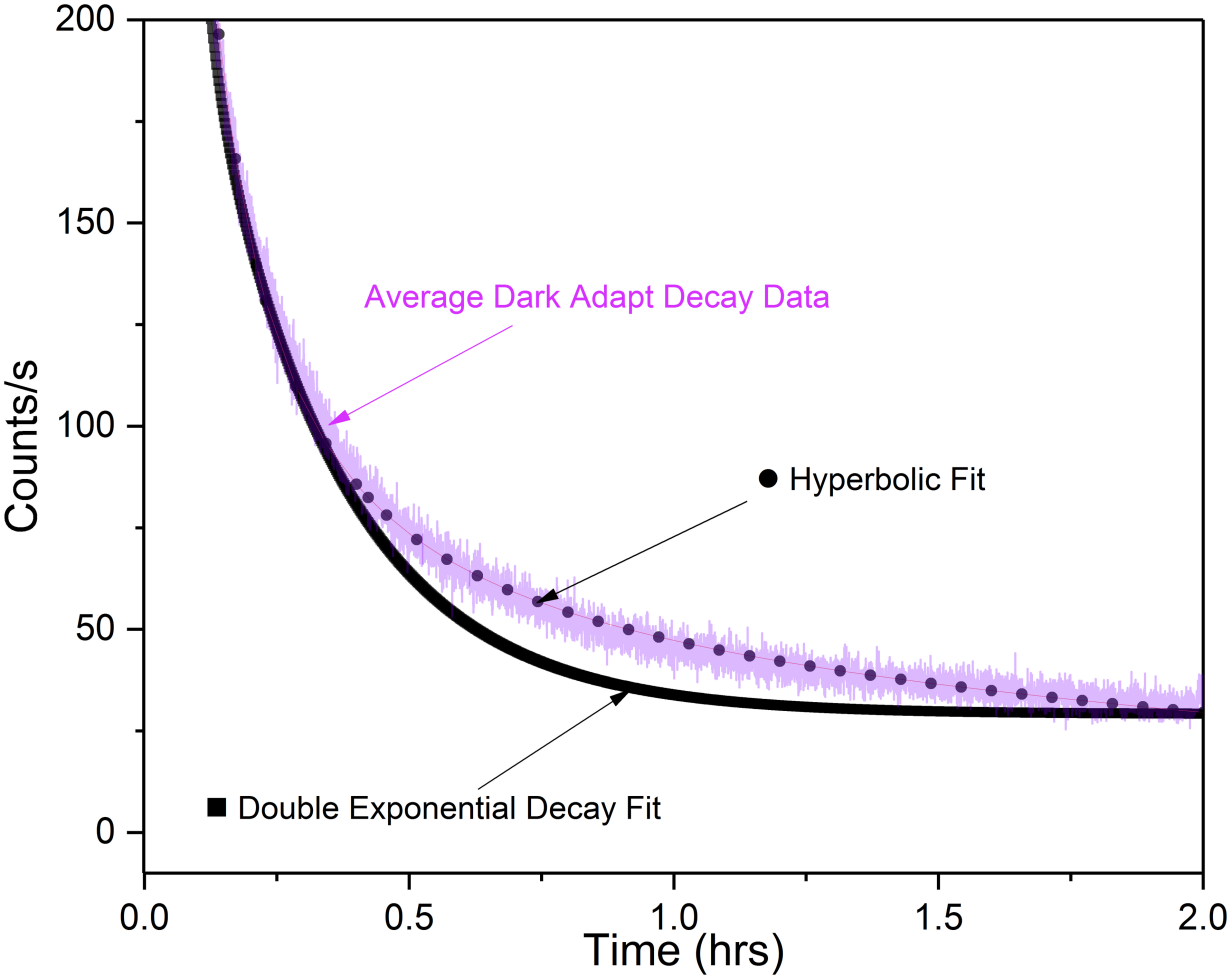
Dark-adaptation decay curve fitting. Comparison of *Spathiphyllum* dark-adaptation decay data with double exponential and hyperbolic curve equations. Magenta line is 9 sample average decay data. Figure shows hyperbolic curve most closely approximates the data.

The hyperbolic function best fit the data (Fig 3). These results correspond with previously reported research that observed the hyperbolic relaxation nature of stimulated biophoton emission both from actinic light [33–35] and through total destruction of a plant [36]. Research involving UPE of anti-cancer herbs stressed through water deprivation, however, reported a double exponential fit though experimental details were limited [37].

### Biophoton wound analyses

To investigate the dependency of wound induced UPE on oxygen; we first characterized leaf luminescence in air at room temperature. Two dark enclosures were eventually constructed. The initial, large enclosure (L) measured 25” x 17” x 21” (~ 5.2 ft^3^) and was placed on the optical table in the dark tent. This large enclosure housed both the PMT and the plant. To mitigate possible effects caused from the PMT cooling fan’s air circulation, a smaller enclosure (S) was constructed that allowed only the PMT lens assembly to protrude into the plant box. Two *Spathiphyllum*s were selected for testing. One spath was used for aerobic (leaves A-C) and anoxic (leaves D-F) wounding experiments. The second Spath was used for the spectral analysis tests. Only leaf (A) was tested in enclosure (L). All subsequent tests were performed in enclosure (S). No more than one test/day was performed and the plant was returned to indirect natural lighting at the end of each day’s testing.

#### Wound-induced biophoton emission (aerobic)

**Fig 4:**
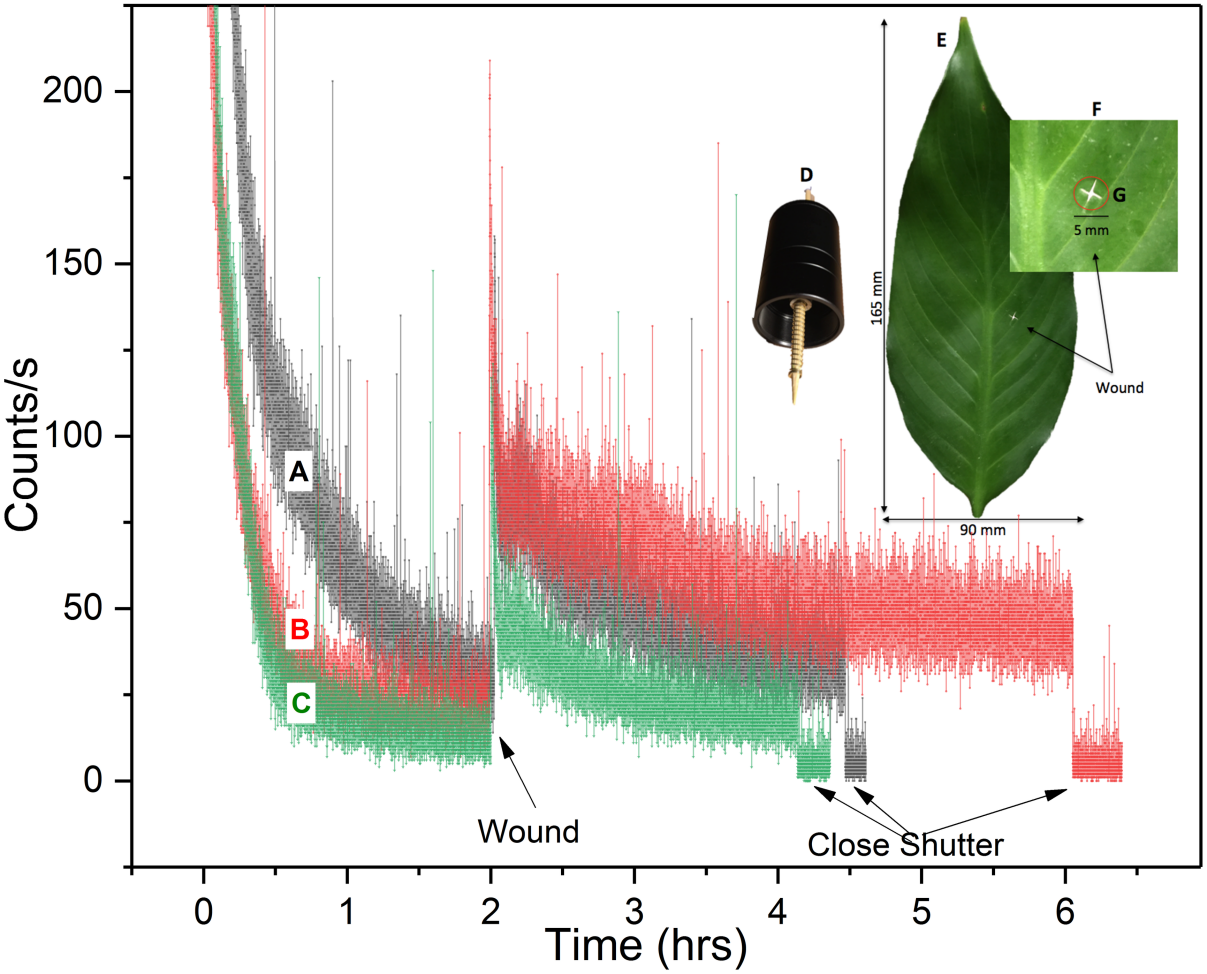
Aerobic wounding UPE. UPE (300–720 nm) of three *Spathiphyllum* leaves (A-C) during aerobic dark-adaptation and wounding at room temperature. (A) Plant in original (large) enclosure. (B-C) Plant placed in smaller enclosure. Photo inset shows wounding apparatus (D) with 3.0 mm bamboo skewer a typical leaf with wound site highlighted (E) and enlargement of the wound (F) showing approximate image area.

Wounding of dark-adapted *Spathiphyllum* leaves under aerobic conditions induced significant biophoton emission. Photon counts increased from about 79–126 cps above the pre-wound basal rate and did not decay back to the pre-wound level even after 4 hours of continuous observation (leaf B). Fig 4 depicts the aerobic wound data recorded for each leaf. The photo inset shows the wounding apparatus and a typical leaf wound site. Observed wounding profiles were similar to those of cucumber and wheat seedlings treated with a toxic agents [34,38].

**Fig 5:**
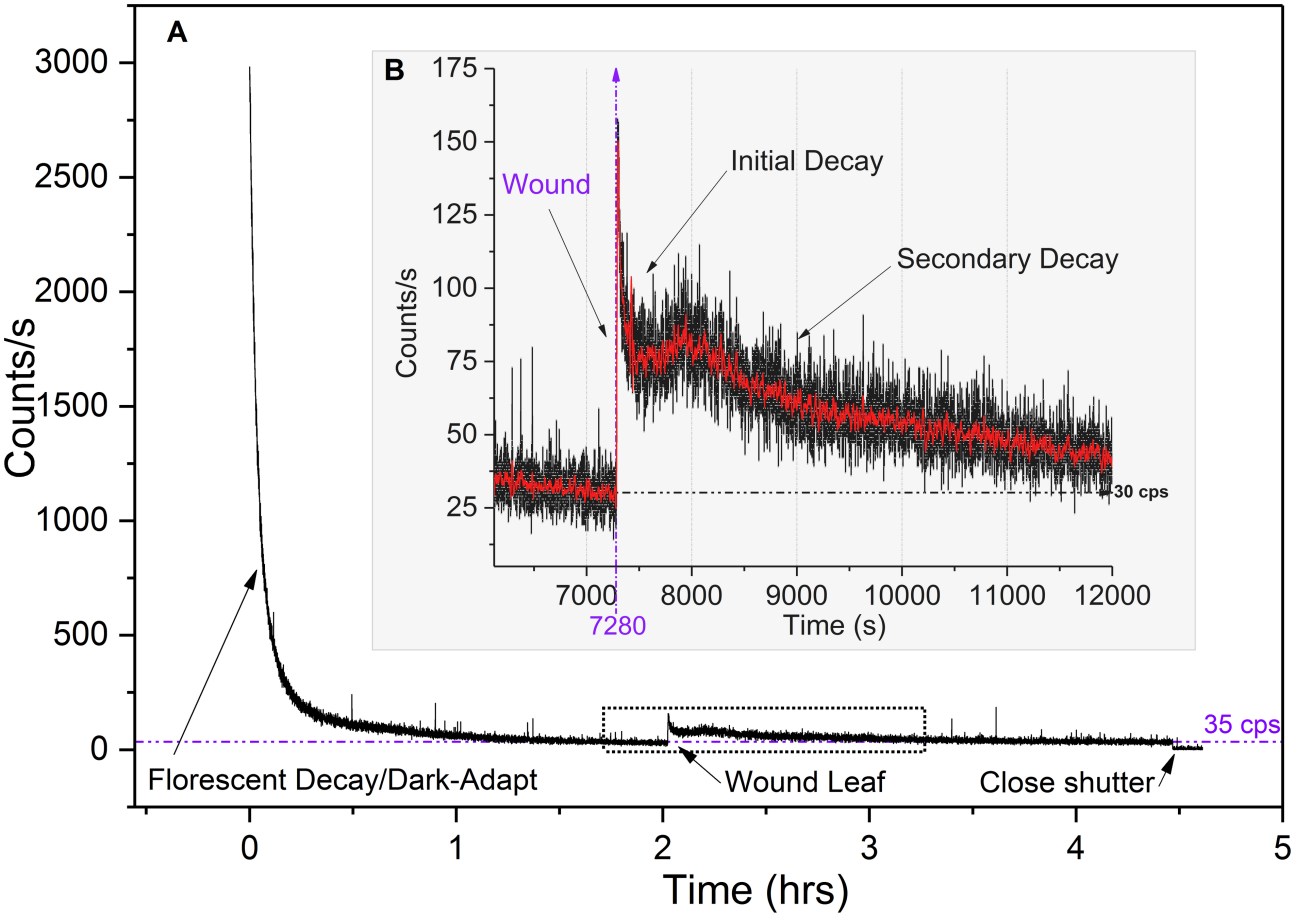
Aerobic wound decay trends. Typical hyperbolic and dual post wound exponential decays (in air). (A) Complete fluorescent decay/dark adapt—wounding profile. (B) Enlarged view of wounding data. Note primary and secondary post wound decays. Red curve is 25-point data smoothing for trend visibility.

Detailed analysis of the aerobic wound induced photon count data revealed two distinct post-wound exponential decays. As expected, the initial decay was observed immediately upon wounding. Secondary decays were observed about 200–300 s after the initial wound decay following a brief plateau in 6 out of 7 measurements. Fig 5 shows the photon count results from one aerobic decay-wound sequence. The enlarged inset highlights the primary and secondary decays.

Both initial and secondary decay curves were fit to a double exponential decay function (see photon counting statistics (PCS) section). The mechanical wounding decay plots differ from typical white or monochromatic light induced excitation decays in that they exhibited no hyperbolic relaxation even after 3.8 hours. We analyzed both pre-wound (post hyperbolic decay) and post wound (secondary exponential decay) photocount data and found both pre- and post-wound photon count distributions closely approximate a Poisson distribution (See PCS section).

It is unclear what biological processes underlay the dual decay phenomena observed. Leaf temperature and humidity did not appear to be significant factors, varying only by 0.2° C and 2–4% respectively during the wounding phase. We suspect that extreme photon counts occasionally observed during the initial tissue wound is attributable to a combination of physical rubbing & destruction of the cell membranes (triboluminescence) coupled with immediate, localized wound induced biochemical processes (oxidative reactions [18]) that have been extensively studied and reported. In addition, chlorophyll molecules are able to reabsorb fluorescence emitted at 680–690 nm within a leaf [39–43]. Therefore, wound site UPE are continually re-absorbed/re-emitted and possibly contribute to the brief secondary rise in luminescence (leading to the plateau) coinciding with the buildup and longer duration lipid peroxidation processes and singlet oxygen/singlet chlorophyll generation cycle. These induced metabolic processes eventually reach a quasi-steady state that is then evidenced in the Poissonian photocount distributions.

#### Wound-induced biophoton emission (anaerobic conditions)

**Fig 6:**
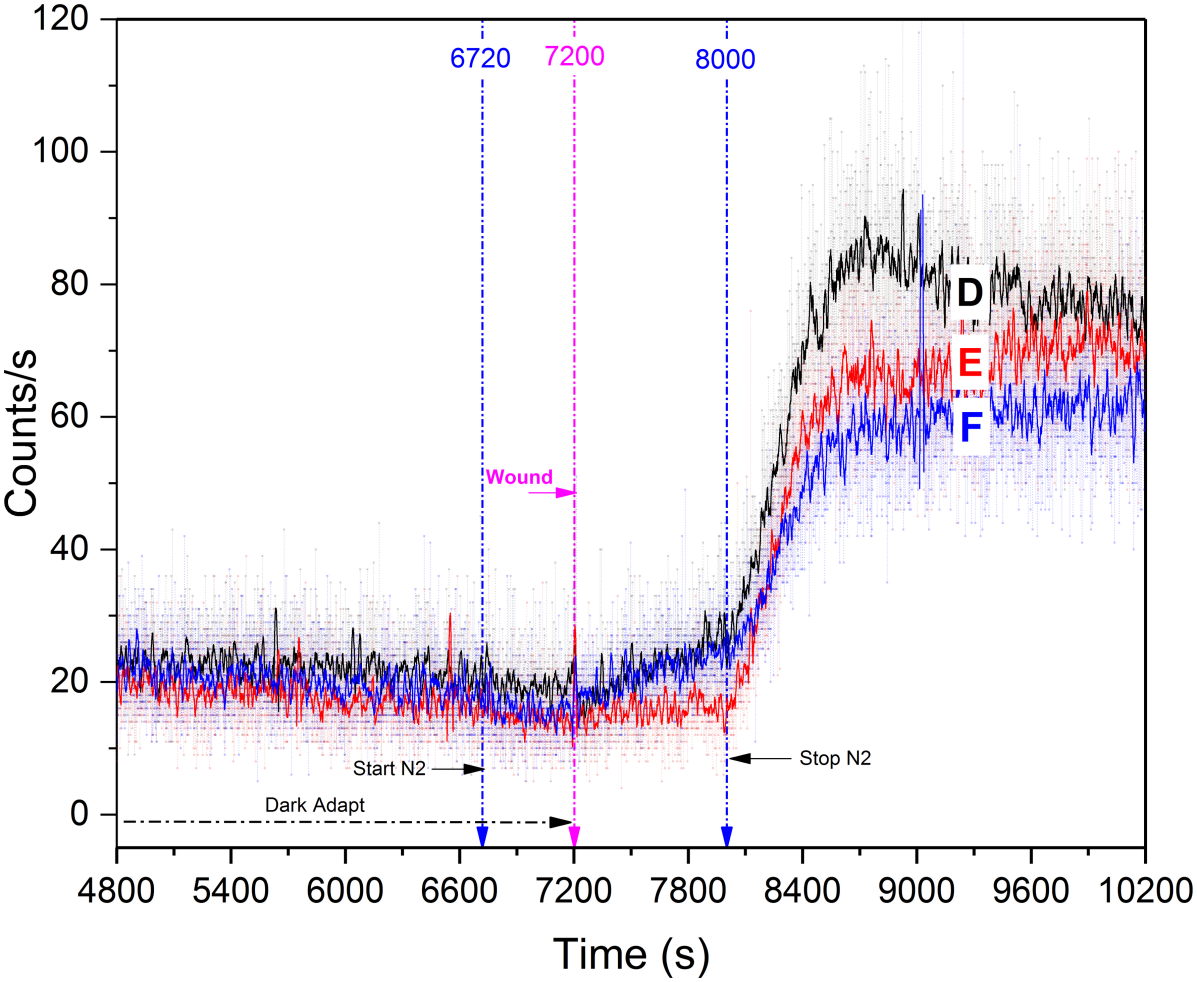
*Spathiphyllum* wounding UPE while under anoxic stress (Nitrogen gas). Moderate increase in photocounts was observed upon anoxic wounding in leaves (D, F). No increase in counts was observed with leaf (E). 25 point smoothing of curve data was used for data visualization.

Nitrogen gas was flowed into the plant chamber at t = 6,700 s and was secured at t = 8,000 s for a total incubation time of 1,300 s (21.67 min.). Three leaves were tested, each on separate days. Nitrogen had no noticeable effect one dark adaptation decay photocounts. Photon counts decreased only by 2–3 cps below the basal rate when nitrogen was introduced 500 s prior to wounding but UPE remained well above the noise floor. At no time did UPE decrease to zero and no transient increase in photocounts was observed when N_2_ was introduced, showing a good agreement to what was as previously reported by other researchers [26,30]. While others have reported significant red light induced UPE from leaves under N_2_ incubation [44], We observed that wound-induced photon emission intensity was greatly inhibited by the anoxic environment, increasing only ~ 8 cps in 2/3 trials. Once nitrogen flow was ceased, biophoton emission increased significantly (peaking ~ 90 cps (Leaf D)) as the chamber was allowed to slowly return to normal aerobic atmospheric conditions (Fig 6). Pre and post anoxic stress linear regression of average wound data was analyzed and is discussed in the PCS section.

The purpose of these anoxic tests was to complement recent research that has shown that chemical inhibition of lipoxygenase directly effects wound induced UPE. We believe we have shown that wounding of leaves *in vivo* in an anaerobic environment restricts the amount of oxygen available for the lipoxygenase catalyzed wounding reactions and ultimately inhibits biophoton emission.

### Wound photon counting statistics (PCS)

We measured photon count statistics of wounded leaves in air and nitrogen in order to compare the observed frequencies with a Poissonian probability distribution as performed in previous studies [33,35,45,46]. The frequencies of which *k*_*n*_ photon counts were registered within a bin (*n*) were obtained using:

$$F(k_{n})=\frac{k_{n}}{k_{t}}$$ (3)

*where the total photon bin counts* is given by

$$k_{t}=\sum_{i=0}^{i\text{max}}k_{i}$$ (4)

Extreme outliers were excluded from the total counts. The Poisson predicted probability distributions of registering (*n*) photons in a preset time interval *(*Δ*t)* was then calculated by:

$$p(n,\Delta t)=\frac{<n{>}^{n}}{n!}e^{-<n>}$$ (5)

where *n* = Bin photon count (*n = 0*, *1*, *2*, *3*, …*max*), <*n*> = mean of all counts within preset time Δ*t*, and Variance = *σ*^*2*^ = <*n*>.

Pre-wound (air & N_2_), wound (air & N_2_), and post wound (air) frequency and Poisson calculations were performed.

### Aerobic PCS

#### Pre-wounding hyperbolic fluorescence/dark-adaptation decay

**Fig 7:**
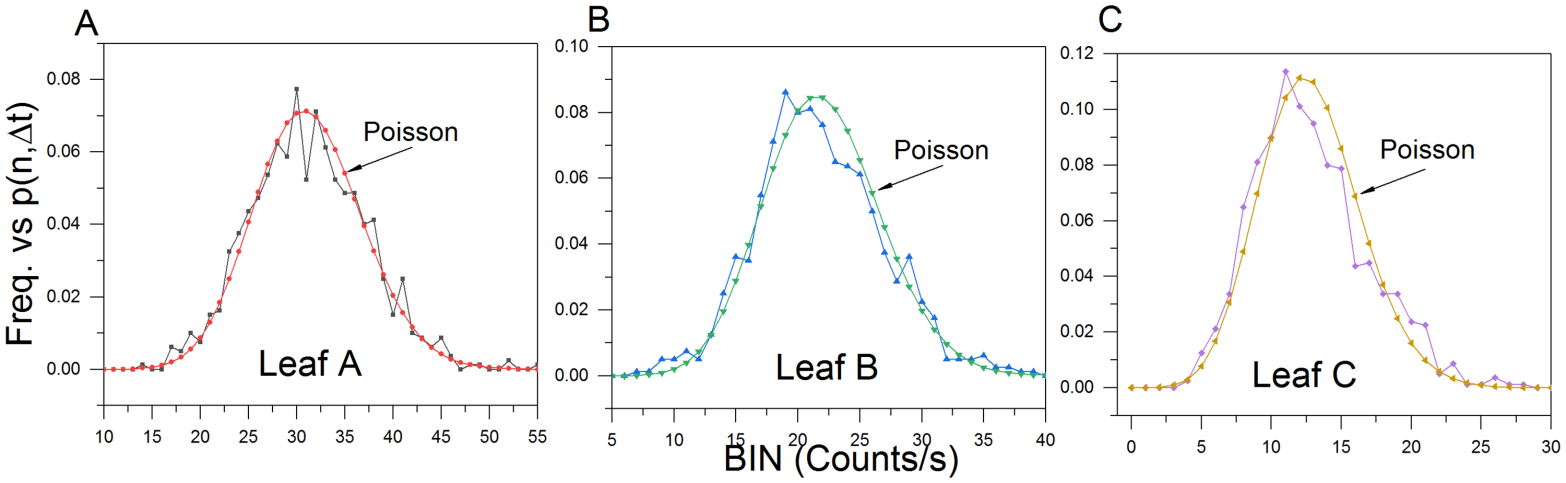
Aerobic pre-wound photocount distributions. Aerobic photocount observed frequencies vs. Poisson predicted distributions (leaves A-C) of the hyperbolic decay data (last 800 s). Observed photon count frequencies closely approximate a Poisson predicted distribution (μ_A_ = 31.25; μ_B_ = 22.03 and μ_C_ = 12.82).

Hyperbolic relaxation decay of normal (photosynthetic) and stimulated (actinic) biophoton emission data has been used to support theoretic claims that a non-local coherent electromagnetic field regulates photon emission [33–35,45–47]. Research has also shown that upon hyperbolic relaxation to a “quasi-stationary state”, the probability *p*(n,Δ*t*) of registering *n* photons (*n = 0*, *1*, *2*, …*)* within the preset time interval *(*Δ*t)* follows a Poissonian distribution [34]. PCS analysis of the last 800s of aerobic hyperbolic relaxation decay curve data closely follows a Poisson distribution (Fig 7).

#### Wound-induced exponential decay

**Fig 8:**
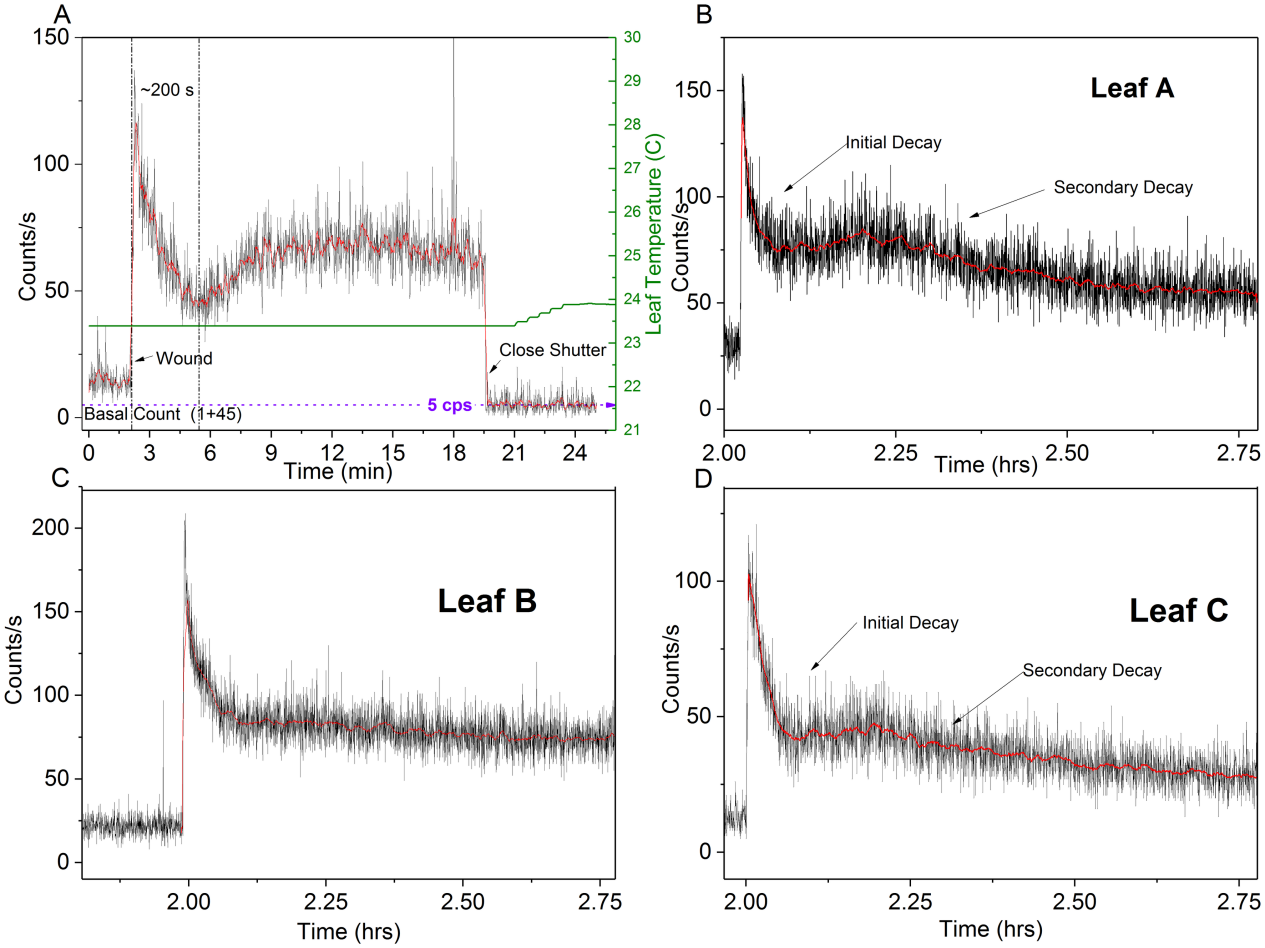
Leaf aerobic wounding photocount comparison. *Spathiphyllum* wounding in air showing distinct peak, secondary rise & subsequent exponential decays (A, B, D). No secondary exponential decay was observed in leaf B (C). Red curve is 10 point smoothing for trend visualization.

In contrast to the coherent excited field (hyperbolic), it has also been suggested that an excited chaotic field decays in accordance with an exponential function [34]. Two distinct wound-induced exponential decays were observed in 6/7 aerobic tests (Fig 8). Each decay was fit to both single and double exponential decay functions.

**Fig 9:**
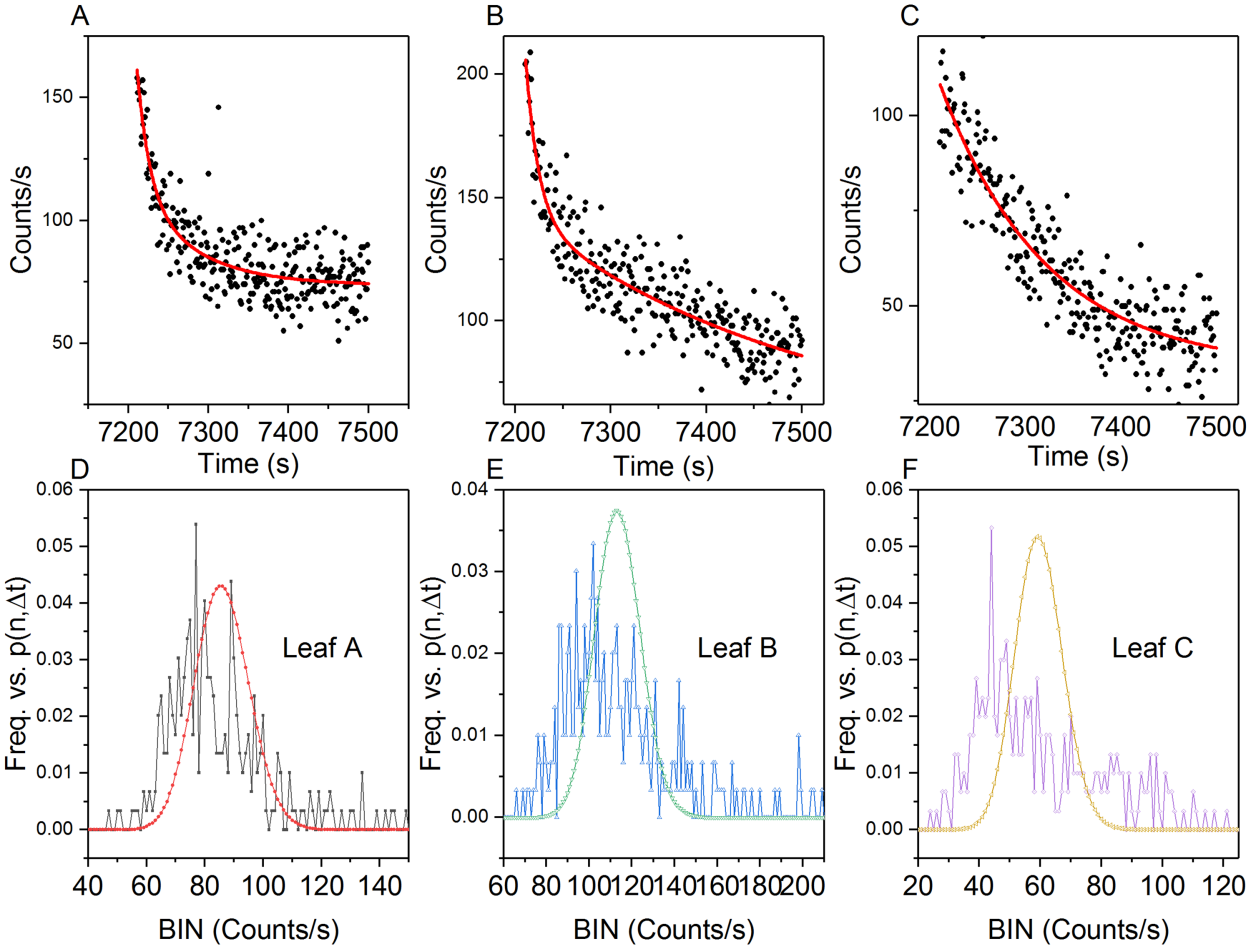
Aerobic post-wound initial decay analysis (300s). Leaves A, B, & C photocounts (A-C) with double exponential decay fit line (red). (D-F) Observed photon count frequency vs. Poisson predicted distribution corresponding to each leaf (μ_A_ = 85.97; μ_B_ = 113.53 and μ_C_ = 59.66).

**Fig 10:**
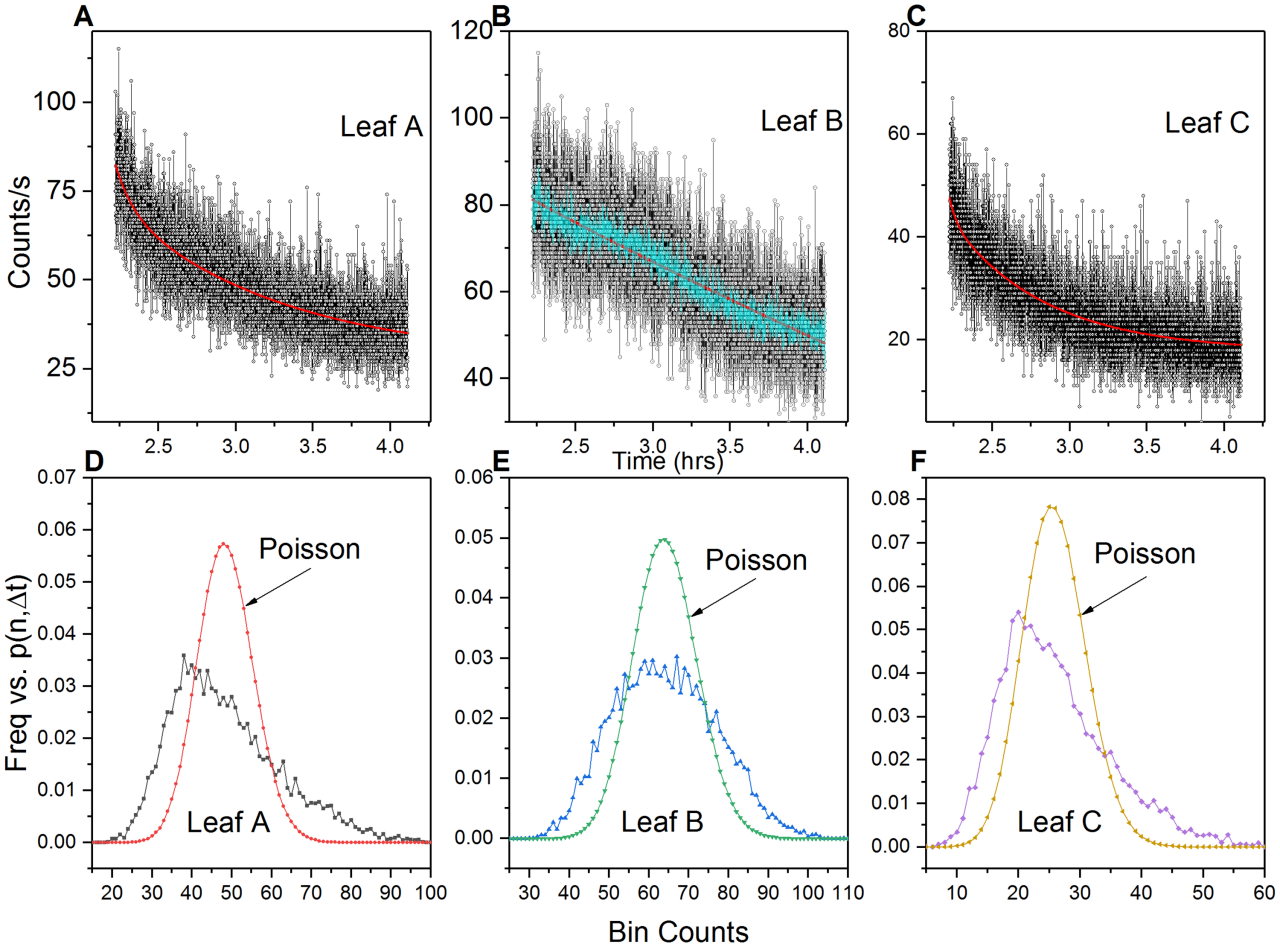
Aerobic post wound secondary exponential decay. Leaves A, B, & C photocounts with double exponential decay fit line (red) (A-C). Leaf B (B) did not display a secondary decay peak. Cyan curve in (B) is 10 pt smoothed to highlight the decay profile. Red lines (A, C) reflect double exponential curve fit. (D-F) Observed photon count frequency vs. Poisson predicted distribution for each leaf (μ_A_ = 48.55; μ_B_ = 64.19 and μ_C_ = 25.90).

The Origin Pro software was unable to converge on a hyperbolic curve fitting function for the data. Comparison models were used to quantitatively assess the curve fitness: Akaike’s Information Criterion (AIC), Bayesian Information Criterion (BIC), and F-tests (p = 0.05). All three models preferred the double exponential decay for the initial decay data (Fig 9A–9C). Two of the three models (AIC: 965 times more likely; F-test: F (3, 6792) = 6.58, p = 0.05) preferred the double exponential decay fit for the secondary decay data (Fig 10A–10C). The second order exponential decay equation is given by:

$$y=y_{0}+A_{1}e^{\left(-(x-x_{0})/t_{1}\right)}+A_{2}e^{\left(-(x-x_{0})/t_{2}\right)}$$ (6)

PCS analysis of the initial and secondary exponential decays showed no Poissonian distribution agreement (Figs 9D–9F and 10D–10F).

**Fig 11:**
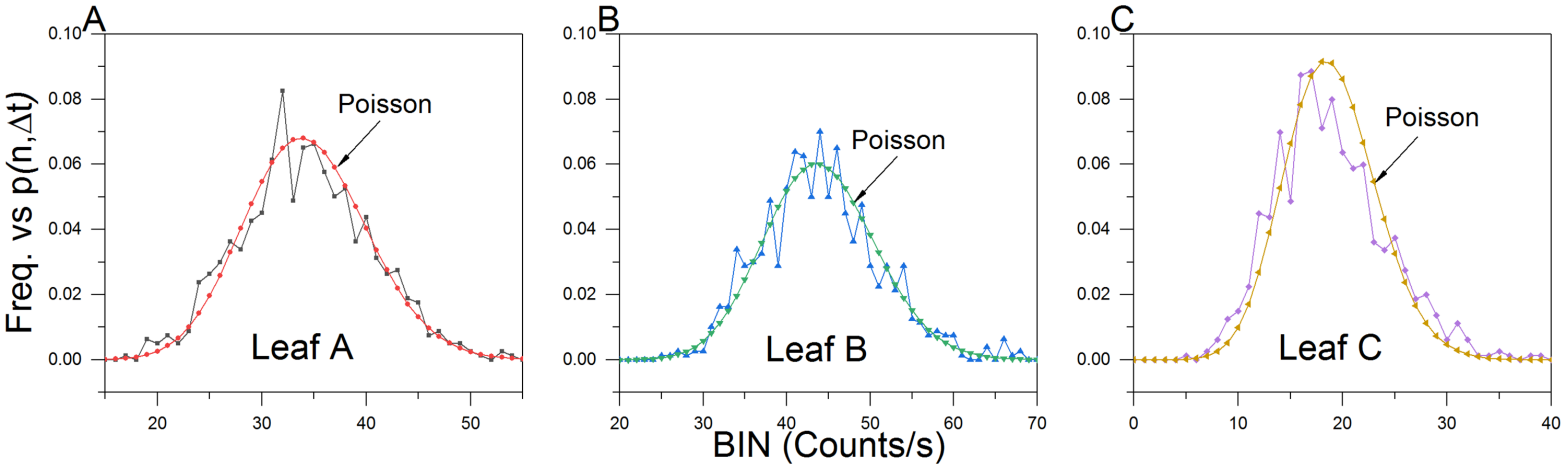
Aerobic post wound secondary decay photocount distributions. Photon count statistics of leaves (A-C) approach a Poissonian distribution upon exponential decay relaxation (last 800 s before closing shutter). (μ_A_ = 34.32; μ_B_ = 44.06 and μ_C_ = 18.90).

End analysis of the aerobic post-wound exponential data (800s before closing the shutter) revealed that the exponential UPE decay did in fact exhibit a Poisson distribution once the wound site reached a “quasi-stable” state (Fig 11).

While we cannot assess the degree of coherence of the plant’s nonlocal field (i.e., macro level), we have observed that the photon counts produced at a single leaf’s wounding site (micro level) do exhibit an exponential decay. We are not suggesting that the leaf or plant as a whole becomes an “incoherent”source, but rather the biophoton emission invoked through highly localized, physical destruction of leaf tissue and resultant wounding response, abruptly change the leaf’s energetic “steady state.” This can perhaps be considered a “chaotic” source of UPE at the level of analysis of the wound site. Further measurements would need to be made to determine if the plant’s overall UPE responds to the micro-level wounding event.

As Shen et al. point out, the existence of a Poissonian photocount distribution is a necessary but insufficient condition for coherence [45]. Distinguishing between coherent and chaotic biological fields is challenged when the measurement time is significantly longer than the coherence time of the system. A 1s gate time was used for all of our photon counting measurements and the distance from the leaf surface (object plane) to the photocathode was approximately 9.4 cm. We do not offer claims for or against the coherence theory. We simply confirm that the “quasi-stable” basal photon counts that follow both hyperbolic and exponential wound-induced biological decays closely approximate a Poisson distribution.

### Anaerobic PCS

#### Post wound analysis (N_2_)

**Fig 12:**
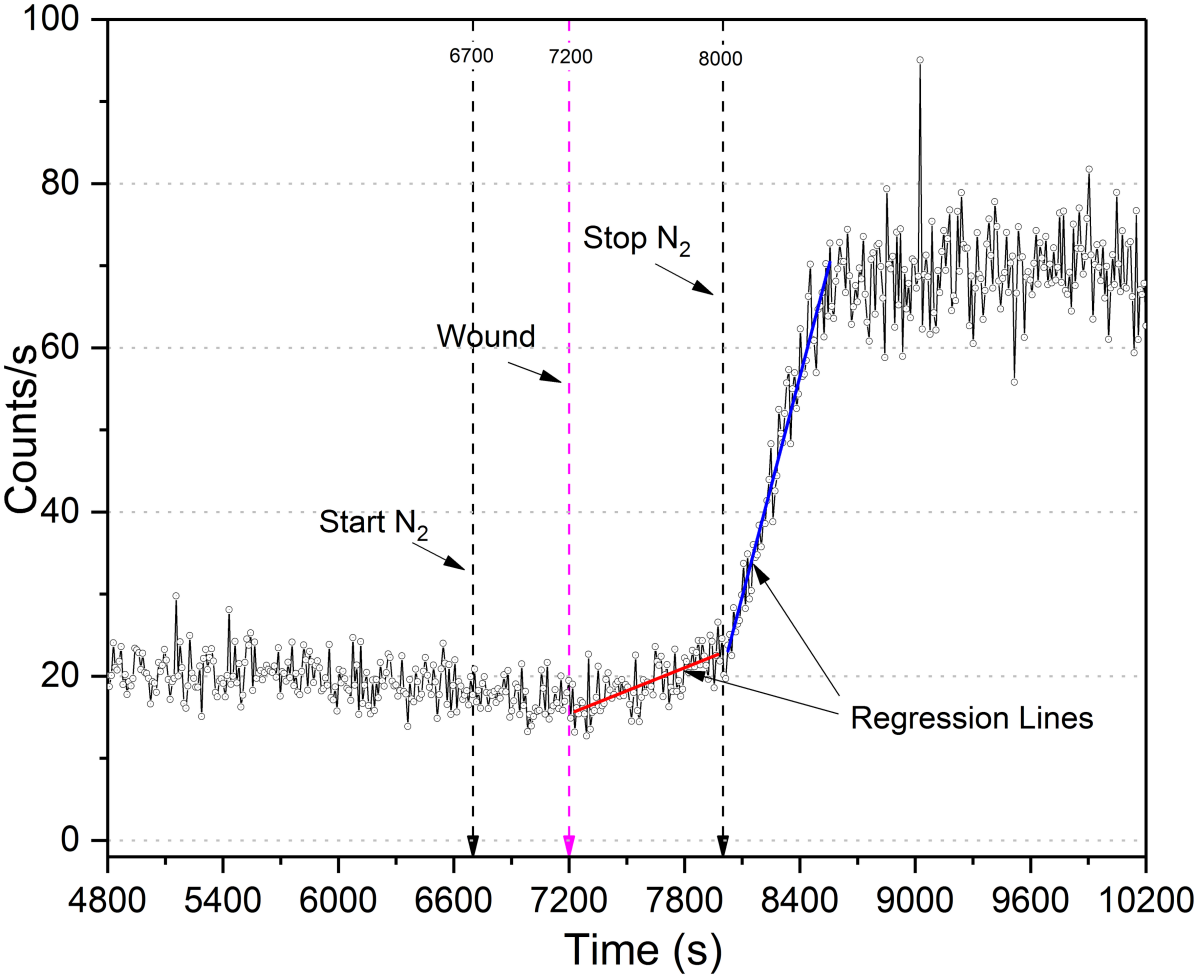
Multiple regression comparison of averaged anaerobic wound data. Three sample average of anoxic wounding data. N_2_ gas introduced at 6,700 s; plant wounded 7,200 s, and N_2_ secured at 8,000 s. The two slopes (red = anoxic, blue = aerobic) differ significantly [F (2, 123) = 416, p = 0.05].

Linear regression of average post-wound anoxic stress leaf photon count data was performed (Fig 12). Photon counts slightly decreased 2–3 cps once the flow of nitrogen was initiated but did not deviate appreciably from the trending decay curve. However, wound induced UPE emission intensity was greatly inhibited by the anoxic environment, increasing by only 8 cps in 500 s of N_2_ incubation. The post-nitrogen phase was statistically significant [F (2, 123) = 416, p = 0.05]; increasing from ~23 to 70 cps (avg) in 540 s.

**Fig 13:**
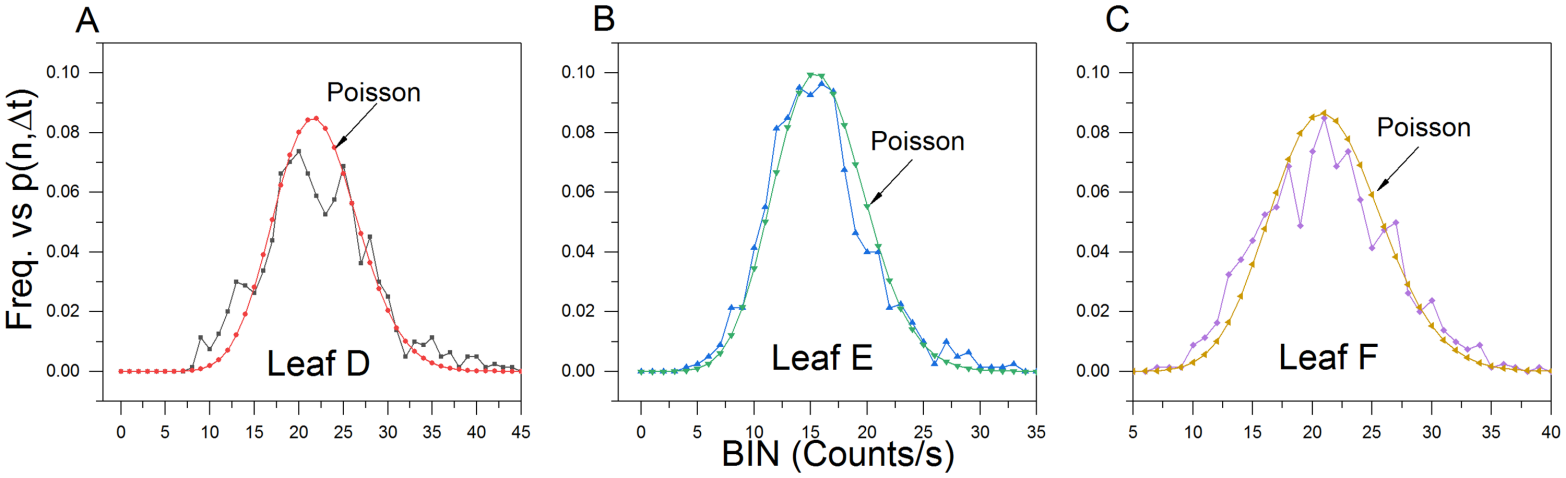
Anoxic wounding photocount distributions. (A-C) Photocount statistics of leaf anoxic wound data. Observed photon count frequencies closely approximate a Poisson predicted distribution (μ_D_ = 22.10; μ_E_ = 15.96 and μ_F_ = 21.34).

PCS analysis of the anoxic wounding data (800 s) closely approximated a Poissonian photocount distribution (Fig 13).

#### Poisson fitness

**Fig 14:**
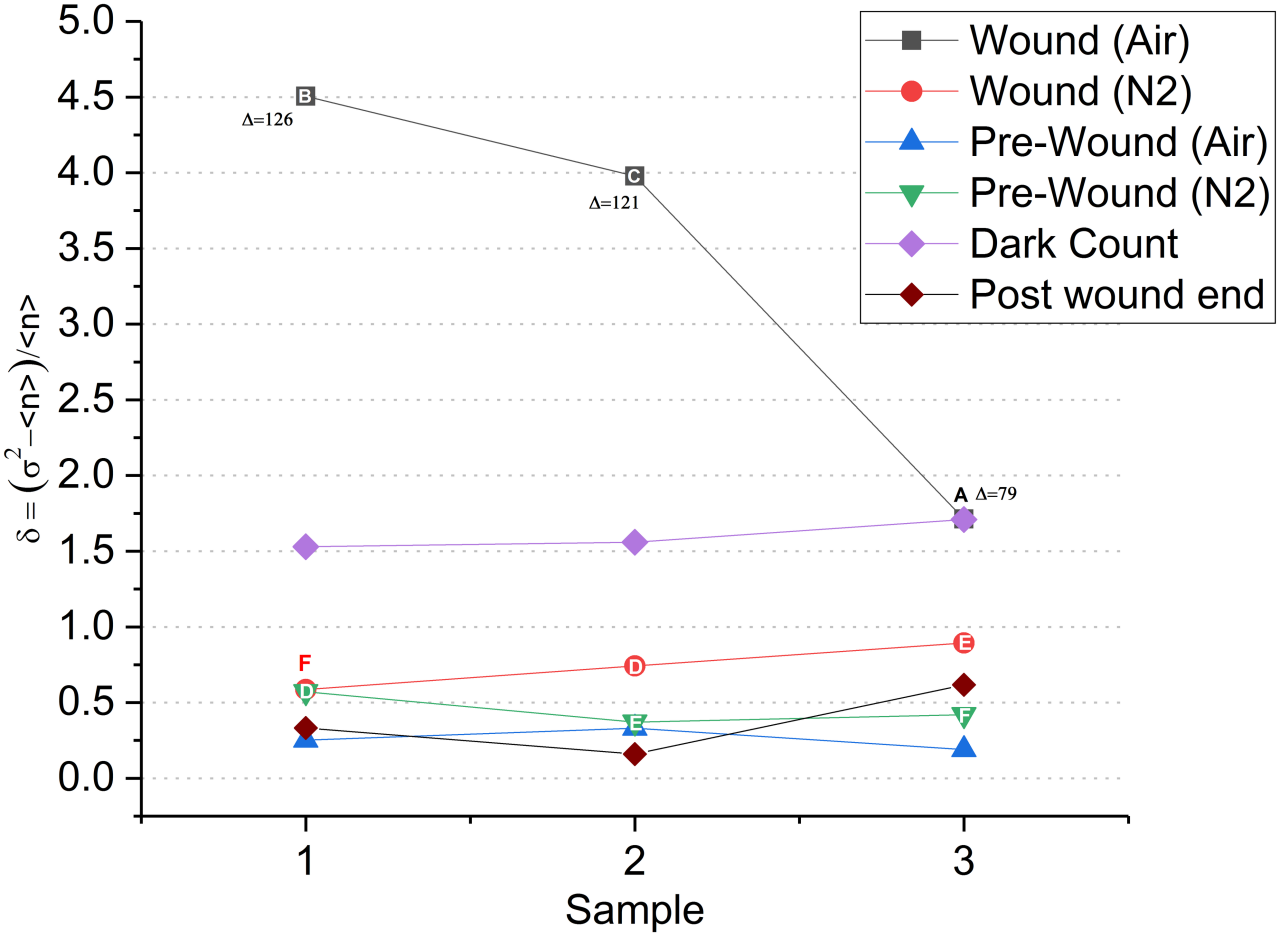
Poisson distribution fitness (δ) of leaf wounding and dark count data. Aerobic wound data surpassed PMT dark counts (2/3 samples). (Δ) Indicates the difference in photocounts from basal—wounding peak and correlates with the degree of divergence from Poisson. Aerobic pre-wound, post-wound end, and anoxic (δ) values show close agreement with a Poisson distribution. (Letters = leaves where indicated).

The degree of fitness with a predicted Poisson distribution, represented by (**δ)**, was calculated using Popp and Shen et al.’s formulation [33,35,45], where (**δ)** is given by

$$\delta =\frac{\sigma^{2}-<n>}{<n>}$$ (7)

where *σ*^2^ is the variance and <n> is the mean. The greater the value of (**δ)** from zero, the less Poissonian the distribution. The (**δ)** was calculated from pre-wound, wound and dark count data (Fig 14). Aerobic wounding produced the greatest deviation from a Poisson fit. The deviation was proportional to the difference between the basal count and initial wound peak. PMT dark count was next highest. All other values fell well below aerobic wounding and random PMT noise data.

### Spectral analysis

#### Pre-wounding fluorescent decay spectral analysis

Room temperature chlorophyll fluorescence results from a combination of related effects (i.e., heat dissipation, dark reduction of plastoquinone, deactivation of Rubisco, and generation of reactive oxygen species (ROS)) [41]. Typically, two chlorophyll emission maxima are observed at room temperature: (i) a red peak around 680–690 nm (attributable to photo system II (PS II)) and (ii) a far-red region around 720–740 nm (emitted by Photo System I (PS I) [41]. While there is a distinct peak maxima at 680 nm at steady state room temperature conditions, the relative emission decreases by ~15% to a 700 nm shoulder before rising to 740–750 nm [39,42,43,48]. Additionally, the reabsorption of chlorophyll fluorescence within whole, intact leaves by PS II is known to result in a strong emission at 685 nm [39,41–43] and 730 nm in isolated spinach chloroplasts [49]. UV-induced blue-green fluorescence (BGF) (Blue: 430–450; Green: 520–530 nm) is also known to be emitted by green leaf plant pigments [50–52]. Chlorophyll-free epidermal cells and major leaf veins are the primary source of green leaf BGF, though the cell walls of green mesophyll cell also contribute to the emission [51].

A second *Spathiphyllum* was used to conduct long duration spectral analysis of the dark-adaptation (2hr.) and wounding phases. Filter spectroscopy measurement details are further discussed in the methods section. Initial filter tests, using the single filter lens configuration (S1 Fig), revealed a slight UPE decay (~10 cps peak) between 388–501 nm (see S2 Fig). This decay was only observed during the first 30 minutes of dark adaptation. We suspect this emission may be attributed to UV-induced BGF since the plant was exposed to sunlight and UV radiation from the overhead white LED lighting prior to dark adaptation. Post fluorescent decay basal photon emission was well above the dark count, and in all cases observed to date, the basal UPE never reached the noise floor even after 8 hours of observation.

**Fig 15:**
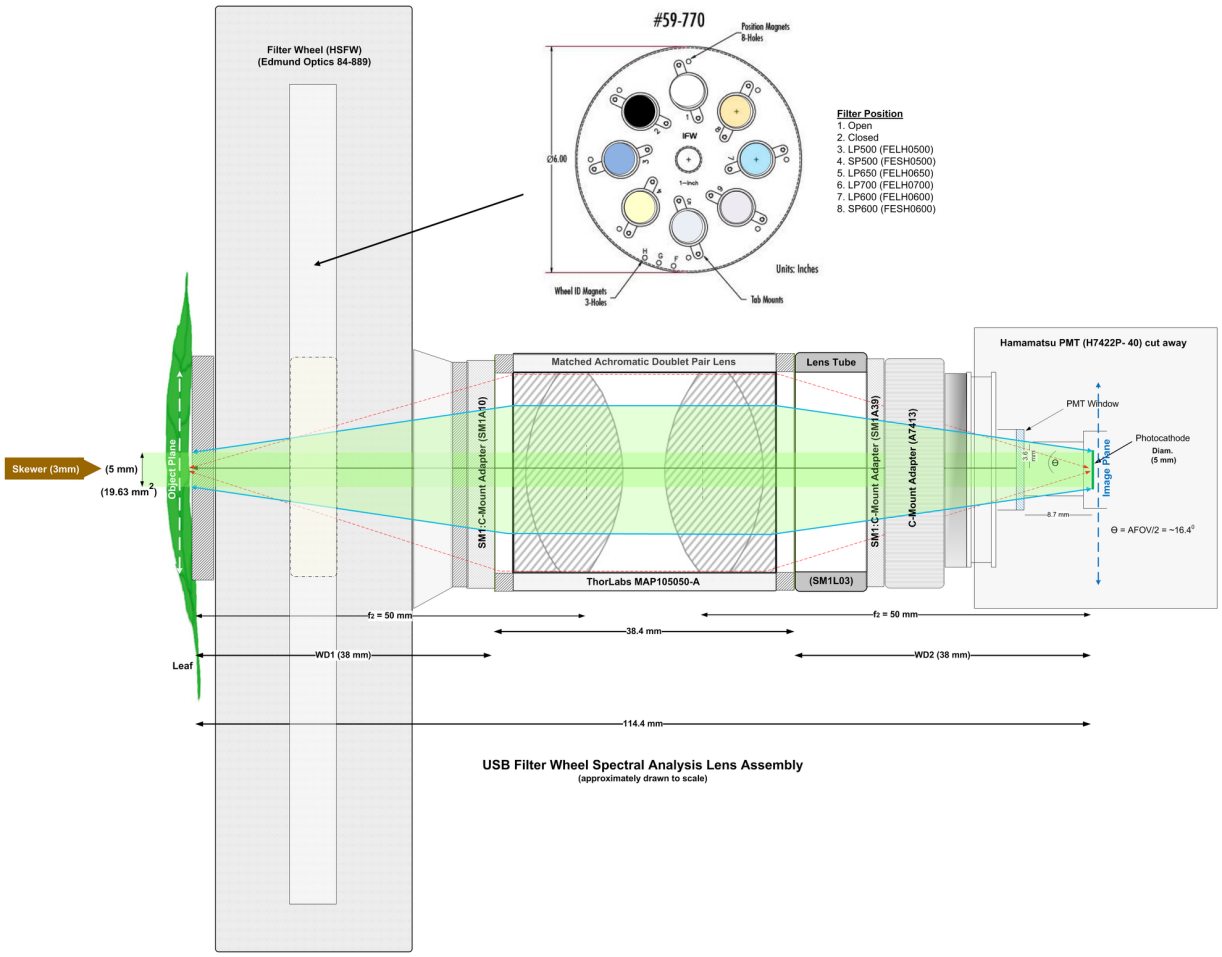
Secondary photon counting lens and PMT assembly. Secondary lens configuration showing matched achromatic doublet pair lens (*f* = 50 mm) and motorized high speed USB filter wheel (HSFW) used in spectroscopy measurements. Filter wheel inset shows filter type for each position. Only positions 1, 2, 5, 6, 7 and 8 were used for the HSFW spectroscopy measurements.

To more accurately assess the leaf UPE spectra, we performed an additional test on a single leaf using a rotating filter wheel lens assembly (Fig 15) configured with filters 3–6.

**Fig 16:**
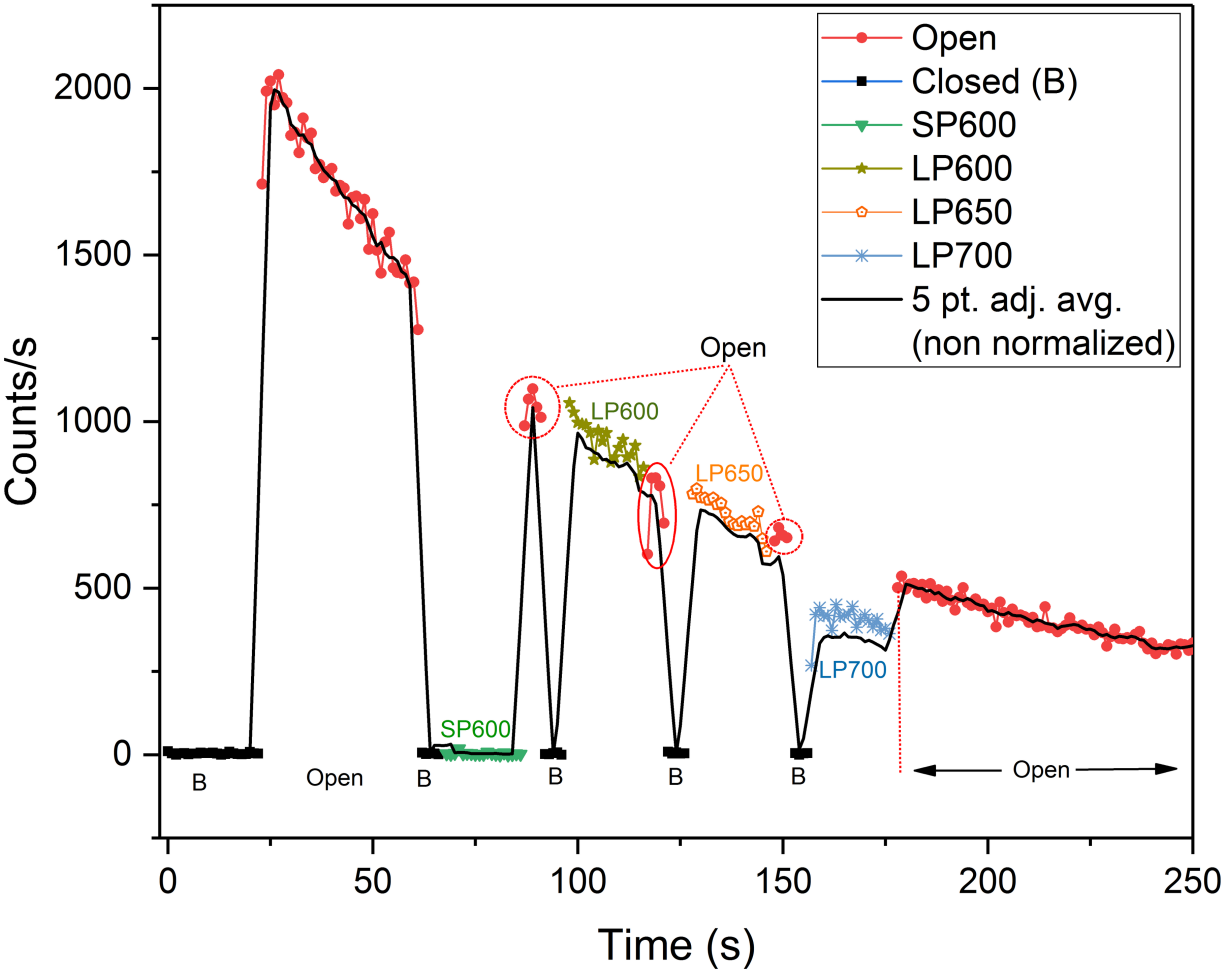
*Spathiphyllum* dark adaptation filter wheel spectral analysis. Leaf UPE during the first 250 s of dark adaptation in air using various optical filters. Solid black line is the non-normalized, 5-point smoothed adjacent average photocount data. Normalized filter data is overlaid for comparison. The decay trend plot is intentionally interrupted by the filter wheel rotation. The filter rotation sequence included a 5 s open (O) and closed (B) phase before each optical edgepass filter measurement (20 s) to better visualize and account for the filter transitions. Data shows no significant UPE above the noise floor from 393–600 nm. Comparison of the LP600 and LP650 plot data reveals that all of the UPE is > 650 nm as the counts are equivalent. The decreased LP700 count data is attributable to the sharp decline of the cathode’s radiant sensitivity at 700 nm vice the filter’s slightly reduced transmittance and thus is inconclusive.

Eight pre-wound filter measurements were performed approximately every 15 minutes starting from T = 60 (Fig 16)—6,300 s (S2 and S3 Figs) (see spectroscopy measurements in Materials and methods section for details). The first 250 s of dark adaption decay data (Fig 16) showed no significant UPE from 393–600 nm. This differs from previously observed data (10 cps, S1 Fig) and may possibly be due to the slightly different optics and greater optical path length of the filter wheel lens assembly. The equivalent LP600 (*N* = 237, *M* = 35, *SD* = 18, *VAR* = 328) and LP650 (*N* = 235, *M* = 35, *SD* = 18, *VAR* = 318) filter data reveals that all of the UPE detected was > 650 nm. Based on these observations it is assumed that this emission is consistent with chlorophyll (PS II), and would be expected during the fluorescent decay process. This does not rule out potential contributing emissions from other excited pigments (550–750 nm) and ^1^O_2_ (634–703 nm) produced through normal metabolic processes that have been reported in this wavelength band [13,53]. The LP700 data, however, is inconclusive as the PMT’s cathode radiant sensitivity declines sharply at 700 nm. This precluded any accurate comparison to the other filter data and limited analysis to spectra below 700 nm. Pre-wound box plot data is provided in S4 Fig.

#### Wound spectral analysis

**Fig 17:**
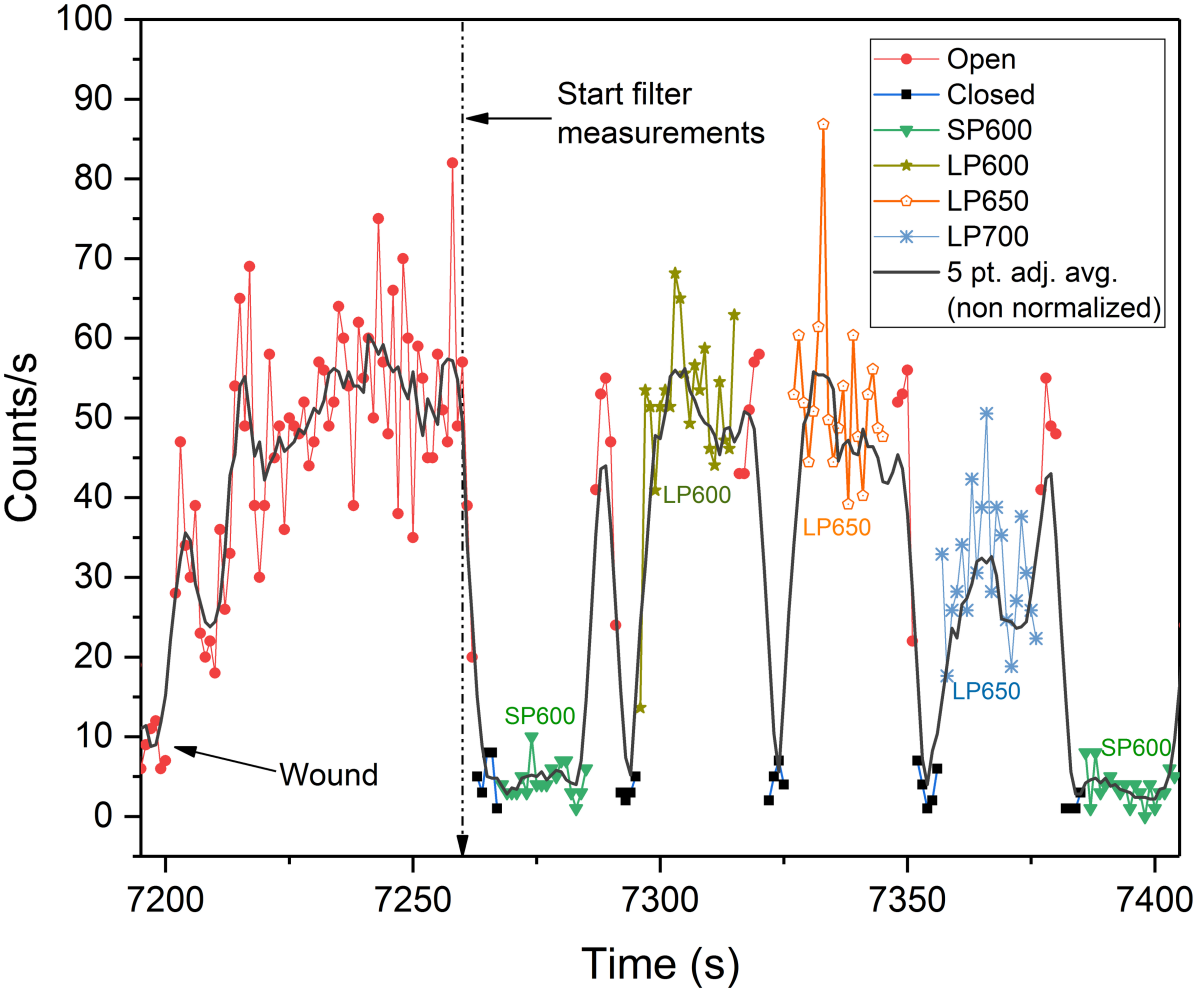
*Spathiphyllum* aerobic wounding filter wheel spectral analysis. Leaf UPE during the first 200 s of aerobic wounding using various optical filters. Solid black line is the non-normalized, 5-point smoothed adjacent average photocount data. Normalized filter data is overlaid for comparison. Leaf was wounded at 2 hrs (7,200 s). The filter wheel measurement sequence was initiated 60 s after wounding. No UPE was detectable below 600 nm. LP600 and LP650 photocounts are equivalent indicating 100% of observed UPE was > 650nm. The reduced LP700 counts are confounded by the PMT cathode’s reduced sensitivity > 700nm.

Spectral measurements revealed a sharp increase in UPE (+ 50 cps) immediately upon wounding (Fig 17). This was in agreement with the findings obtained from the single filter tests previously conducted (S5 Fig). Continuous filter wheel measurements (*N* = 4) commenced 60 s after wounding with a final measurement taken at the experiment’s end (~4.4 hrs) (S6 Fig).

These results showed that all of the detectable UPE was > 650 nm. Statistical comparison of the LP600 (*N* = 99, *M* = 41, *SD* = 11, *VAR* = 117) and LP650 (*N* = 97, *M* = 42, *SD* = 10, *VAR* = 109) filter data shows the data are nearly equivalent (similar to the pre-wounding results). Though UPE was observed from 700–720 nm, the reduced photon counts of LP700 filter, were most likely to be an effect of the reduced cathode radiant sensitivity > 700nm. This prevented any comparative quantitative assessment. Filter wheel statistical data are summarized in S1 Table and in S4 Fig box plots.

Our optical system was unable to detect any significant wound-induced UPE (< 600 nm) above the noise floor even though UPE spectra has been reported in: lipoxygenase-catalyzed linoleate oxidation reaction emissions (450 nm and 550 nm; shoulder at 630 nm) in a cuvette solution [19], triplet excited carbonyls (350–550 nm) [13,53]; (450–550 nm) [16]_;_ dimol emission of singlet oxygen (460 nm and 510 nm) [12]; and in oxidized proteins (490–580 nm) [15].

These wound-induced UPE spectral observations contribute to reported research findings in *Arabidopsis* (100% UPE > 695 nm) [15,18]; 700–750 nm [17] and in Geraniums (89% UPE 600–1000 nm) [54]. In addition to singlet excited chlorophyll emissions (670–740 nm) [13,53], other potential emission sources include singlet excited pigments (550 nm—750 nm) and dimol emission of singlet oxygen (634 nm and 703 nm) [12,13], and oxidation of linolenic acid (640–695 nm) [15].

## Materials and methods

### *Spathiphyllum* test conditions

Plants consisted of two identical *Spathiphyllum* cultivars also referred to as leaf spathe or Peace Lily, purchased from different local floral merchants. The actual cultivar type [55] was unknown but it was not of the petite variety. *Spathiphyllum* is an indoor, low-light foliage plant with amphistomatic leaves [56]. The “Spaths” were selected for their small size and large leaves. This enabled measurements to be performed *in vivo* and allowed the leaf to completely cover the lens aperture. One spath was used for both the aerobic testing (leaves A-C) and the anoxic tests (leaves D-F). The second spath was used for the spectral analysis tests. The plants were not blooming at the time the anoxic and single filter tests were performed. However, the second spath was beginning to bloom during the filter wheel spectrum measurements. The plants were typically kept under indirect sunlight and brought to the lab only on the morning of testing. Once in the lab, plants were exposed to routine laboratory overhead white LED lighting before being placed in the dark enclosure. Non-actinic indirect light (3 W green LED bulb) was used in the dark tent to provide the researcher enough ambient lighting to safely place the plant in the dark enclosure while minimizing light stimulation of the leaf. The leaf was secured to the lens aperture with the upper epidermis facing the photocathode. The total enclosure process preparation time averaged approximately 10–15 min. Wounded leaves were only measured once. A new, unwounded leaf was selected for each test (except for the filter wheel spectrum measurement which could not be avoided). The enclosure was then closed and sealed with 2” AT205 black aluminum foil tape (Thorlabs, Newton, NJ, USA) and the wounding assembly was emplaced.

#### Plant and dark tent test enclosures

**Fig 18:**
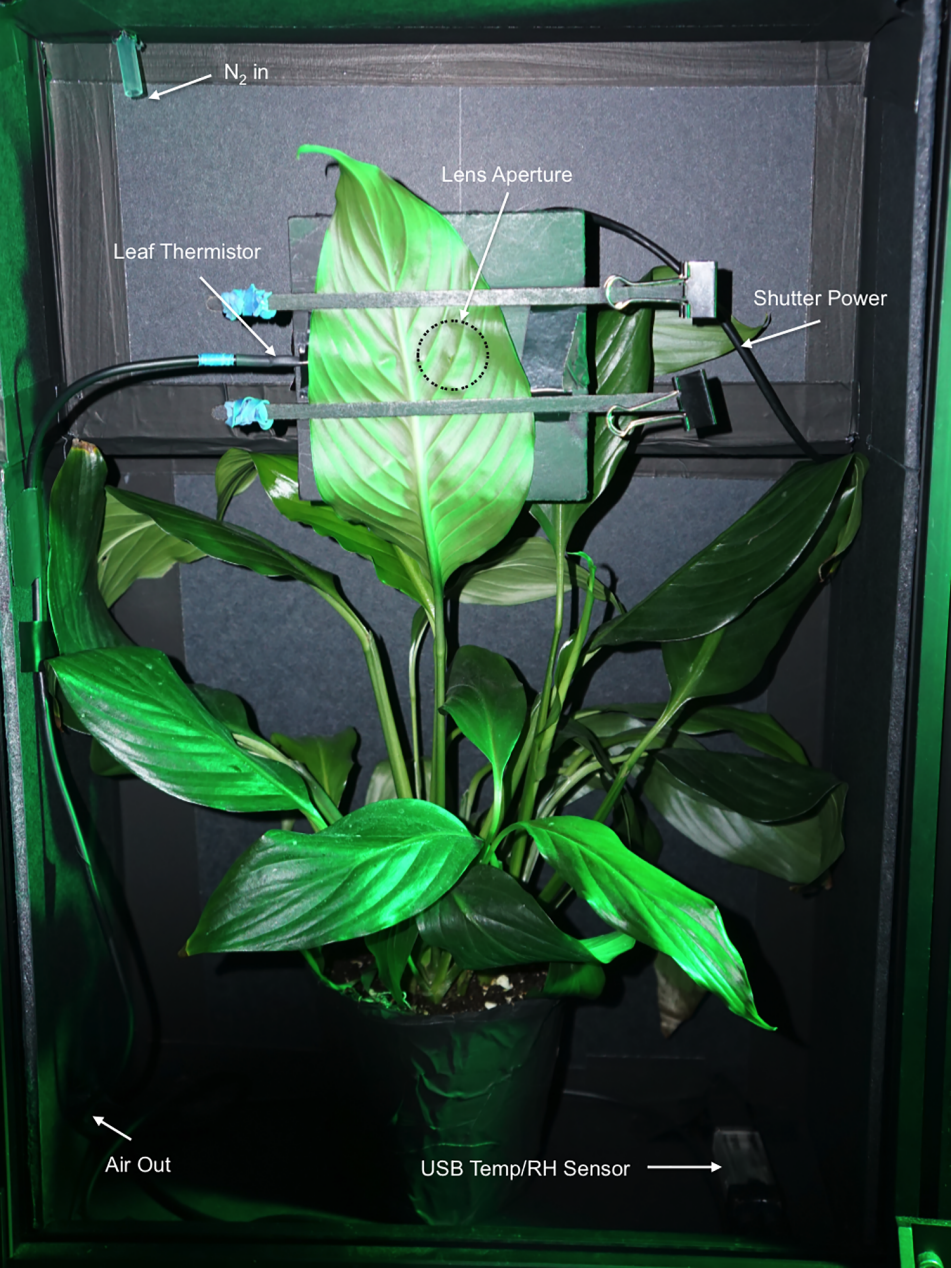
Plant test enclosure. *Spathiphyllum* in plant enclosure (S). Shown with single filter lens assembly and front panel removed.

The plant enclosure (vol. 0.019 m^3^) was constructed with 5 mm thick, black plastic-coated TB4 foam hardboard (Thorlabs, Newton, NJ, USA) (Fig 18).

Continuous temperature and relative humidity data was collected during each measurement. A USB temperature and relative humidity (RH) data logger TSP01 (Thorlabs, Newton, NJ, USA), with the status indicator LED removed, was placed inside the plant dark enclosure. The TSP01 monitored the plant enclosure temperature and relative humidity (%). Two thermistors were connected to the TSP01 to additionally measure (1) leaf temperature at the lens tube aperture and (2) dark tent room temperature (connected outside the dark box enclosure).

A small square piece of foam poster board with a 25 mm hole was affixed to the lens tube assembly and used to hold the leaf in place. A 25 mm hole was cut in the front panel of the plant enclosure and rigged with a lens tube wounding apparatus.

The wounding apparatus was constructed with 25 mm lens tubes, a spring, and a 3 mm bamboo skewer. A 3 mm hole was drilled through the lens caps on each side of the lens tube assembly to guide the skewer to puncture the leaf near the center of the object plane. The spring allowed the skewer to (1) remain retracted while the leaf was positioned to avoid premature plant wounding and (2) to allow it to return to the pre-wounding position. The wound apparatus was operated manually by hand.

**Fig 19:**
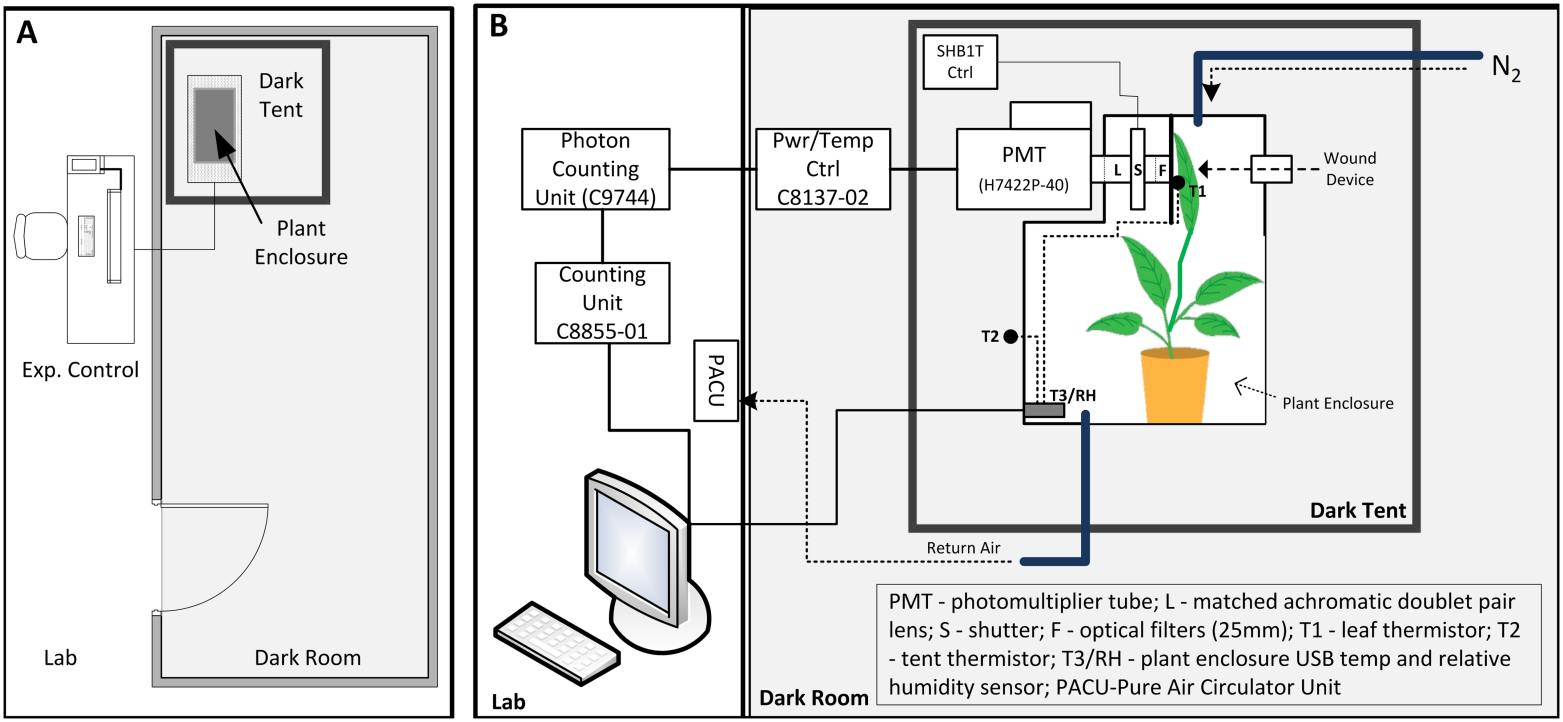
Experiment layout and test equipment. (A) Overview of lab and dark room. (B) Photon counting experiment set up.

The plant enclosure was located inside a dark tent, GGT 5x5 (Gorilla Grow Tent, Inc., Santa Rosa, CA, USA) measuring 1.5 m x 1.5 m x 2.1 m. The inside of the dark tent was lined (4 sides and ceiling) with blackout curtains (Textron) to cover the tent’s internal silver reflective coating. The supplied reflective floor mat was inverted so that the flat black side faced upward. The entrance to the tent was not zippered shut but remained completely covered by overlapping blackout curtains. This allowed easy access. The tent was housed inside the lab’s dark room (6.5 m x 2.8 m x 3.6 m), which was painted black, and the door to the room was covered with an additional blackout curtain to further reduce external light. The overall lab and experiment set up is provided in Fig 19.

### Tests involving nitrogen (N_2_) gas

A cylinder of nitrogen gas was placed just outside of the dark tent and ¼” fluoropolymer tubing was run into the tent via a side access vent. When signaled by the synchronization trigger, the researcher entered the dark room and manually turned on/off the N_2_ gas. Gas flow was estimated to be ~ 10 liters/s. The air return line of a Pure Air Circulator Unit (PACU) (Thorlabs, Newton, NJ, USA) was connected to the plant enclosure to assist in evacuating room air (4 liters/s) as N_2_ gas filled the box. The PACU was not used in closed loop mode. The wounded leafs were exposed to N_2_ for 21.3 minutes (t = 6,720–8,000 sec). N_2_ and the PACU were then secured and the plant box was allowed to return to normal atmospheric concentrations.

It was anticipated that the PACU would be able to provide clean, dry, VOC-free air in a closed loop mode to the plant enclosure. Aerobic experiment data revealed the combined ambient humidity and large volume of the plant enclosure quickly saturated the PACU desiccant filter. Subsequent spectral analysis tests did not employ the PACU in closed loop mode. The PACU was only used to assist in air removal of the plant enclosure during the anoxic experiments.

### Ultra-weak photon emission measurement

#### Highly sensitive PMT

One-dimensional photon counting was performed using a low noise H7422P-40 (Hamamatsu Photonics, K.K., Iwata City, Japan) photo sensor module (or photomultiplier tube (PMT)). The PMT has a 5 mm gallium arsenide phosphide (GaAsP) photocathode with a spectral response in the visible range of 300–720 nm and 40% quantum efficiency (QE) at 580 nm. The PMT was configured with a stock heat sink, fan, and Peltier thermoelectric cooling element cable of maintaining the photocathode at a temperature of 0° C. Consistently measured dark count with the lens cap off in the dark room was 5 counts per second (cps) (N = 10, n = 1,000, <n> = 4.54, σ = 3.58). The PMT was connected to a C9744 amplifier and pulse shaping unit (Hamamatsu Photonics, K.K., Iwata City, Japan) and then to a C8855-01 photon counting unit (Hamamatsu Photonics, K.K., Iwata City, Japan) and connected via USB to a MAC (iMac, MacBook Pro) running VMware, Windows 7E (Fig 19). The discrimination level of the C9744 was manually set to 80 mV. The C8855-01 employs dual counters and is capable of measuring input signals with no dead time.

The PMT was equipped with an anti-reflective (AR) coated, matched achromatic doublet pair lens. A matched achromatic doublet pair lens consists of two identical achromatic doublet pairs housed (in this case) in an SM1 (1.035”-40) lens tube. When combined they create a finite conjugate configuration where the object and image are a finite distance from the lens pair. This creates an image relay system as opposed to an image magnifier. Two such lenses were used: (1) MAP104040-A for the four individual leaf measurements and (2) MAP105050-A (both from Thorlabs, Newton, NJ, USA) for the filter wheel spectroscopy experiments. The lens system magnification was 1:1 with focal lengths (f_1_ & f_2_) of 40 mm and 50 mm respectively. Similarly, the working distances (where WD_1_ = WD_2_) of the MAP104040-A and MAP105050-A were 28 mm and 38 mm respectively. The object and image planes were located at the lens WD. In this arrangement, the object image height is equal to the photocathode diameter of 5 mm, equating to an object image area of 19.63 mm^2^. The angular field of view (AFOV), (44.96 ^o^ for the MAP-104040 and 32.8 ^o^ for the MAP-105050 lens assemblies), was constrained by both the PMT housing and the photocathode size, which was recessed ~ 16.3 mm from the front face of the PMT module. (See Figs 1 and 15 for a complete description of the lens assemblies).

An electronic shutter SHB1T (Thorlabs, Newton, NJ, USA) was inserted between the lens tube and the matched achromatic doublet pair. The shutter controller was inside the dark tent and was controlled manually. The SHB1T’s LEDs were blacked out with a combination of black liquid tape and black aluminum tape to prevent any light leakage. The shutter was removed for the filter wheel spectroscopy measurements and a black aluminum 25 mm disk (filter wheel position #2) was used instead.

#### Spectroscopy measurements

One of the two Spaths was dedicated to the spectral analysis experiments. Two types of spectral analysis tests were performed. The first set of tests consisted of four separate, long duration (~3.5–5 hr) measurements using a single edgepass filter (see Fig 1). The optical filters (Thorlabs, Newton, NJ, USA) are listed in Table 1. A new, healthy, unwounded leaf was used for each of these tests. Leaf spectral response was measured continuously throughout the dark adaptation and wounding phases using filters 1, 2, 5, and 6 (see Table 1).

**Table 1:** Edgepass filters used for spectral analysis.

Filter	Filter Type/ (Model #)	Cutoff (λ)	Overall Band (λ)	Avg. Transmittance
1	Short Pass (SP500) (FESH0500)	500 nm	388–501 nm	93.72%
2	Long Pass (LP550) (FELH0550)	550 nm	546–720 nm	94.70%
3	Short Pass (SP600) (FESH0600)	600 nm	393–600 nm	95.73%
4	Long Pass (LP600) (FELH0600)	600 nm	596–720 nm	95.37%
5	Long Pass (LP650) (FELH0650)	650 nm	647–720 nm	94.43%
6	Long Pass (LP700) (FELH0700)	700 nm	700–720 nm	85.04%

The second spectral test employed a USB controlled, 25 mm, 8-position High Speed filter wheel (HSFW), Model 84–889 (Edmund Optics, Barrington, NJ, USA) and the MAP105050-A achromatic doublet pair lens (Fig 15). A single previously wounded leaf was used for this measurement though the new wound site was targeted on a healthy portion of the leaf opposite the midrib of the original wound that was over 60 days old (S7 Fig). Thirteen measurement sequences were recorded over 4.5 hrs using filters 3, 4, 5, and 6 (see Table 1). Spectrum was measured with each filter for ~ 20 sec. The open (O)-no filter and (B) black—black aluminum disk positions were used to visually identify filter positions in the data (duration 5 sec). The filter wheel measurement sequence (total 130 s): | (O) | (B) |SP600 | (O) | (B) | LP600 | (O) | (B) | LP650 | (O) | (B) | LP700 | (O) | (B) | was repeated every 15 minutes (from t = 60 s to t = 6,300 s; N = 8) until wounding. The filter sequence was then repeated four times continuously beginning 60 s after wounding (N = 4). The last measurement was recorded at ~4.375 hrs (N = 1). Dark counts during these tests were obtained with the “black” filter position. No shutter was used with this lens configuration. The leaf and enclosure temperatures (23.7° C / 21.0° C) respectively, were stable within 1° C from wounding through the end of the experiment with relative humidity (RH) increasing only 3% over the same time period from 60.9–63.9%.

Filter data was normalized by dividing the measured photon counts by each filter’s average transmission (%) value (manufacturer provided) for the respective wavelength band (see Table 1). Filter movement was controlled manually from in the lab via supplied software (OPTEC, Inc., v2.0.9) using an audio timing sequence created in Audacity (see Software).

#### Software

The C8855-01 supplied software was used to control the PMT and record the photocount data. Photon counting data (.csv) and temperature log files (.txt) were saved and uploaded into Origin Pro 2017 and Microsoft Excel (2011) for post-experiment data analysis as required. MS Excel was used to further analyze Origin Pro photon counting statistic data to calculate bin frequencies and predicted Poisson distribution data. The spreadsheet formula “POISSON.DIST(x, mean, FALSE)” was used to calculate the predicted Poisson distribution where in our case x = Bin (or “expected count”), mean = mean, and FALSE = the cumulative value to return the probability mass function that the number of events occurring will be exactly x. Calculated values were then imported back into Origin Pro for graphing and further analysis.

The open source sound editing software Audacity (v 2.1.2) was used to provide the experimenter warning tones to synchronize the controlled timing of manual wound inducement and filter wheel positioning.

## Conclusions

Long duration ultra-weak photon emission of dark-adapting whole *Spathiphyllum* leaves (*in vivo*) was continuously measured, at room temperature, using a low noise PMT in a light trap environment. Leaves were mechanically wounded after two hours of dark adaptation in aerobic and anaerobic conditions.

Under aerobic conditions, the dark adaptation photoemission exhibited a hyperbolic decay. Subsequent mechanical wounding of the leaves induced significant biophoton emission. Photon counts increased from about 79–126 cps above the pre-wound basal count rate and did not decay back to the pre-wound level even after several hours of continuous observation.

Post-wound aerobic photon counts displayed two successive double exponential decays. The primary UPE decay resulting from the leaf’s puncture might be attributable to a combination of previously reported oxidative (chlorophyll luminescence) effects as well as triboluminescent contributions resulting from the violent rupture of the leaf’s cellular structures, rubbing of the wounding device on the aluminum lens tube, or a combination thereof. The secondary rise, exhibiting much lower intensity and subsequent photoemission decay is more likely to be the result of a combination of reabsorption of wound-induced UPE as well as the buildup and propagation of longer duration oxidative processes associated with the plant’s healing processes.

Spectral analysis of both the aerobic pre-wound and wounded leaves revealed ultraweak photon emission predominates at wavelengths longer than 650 nm, while minute (10 cps) UPE was detected at wavelengths shorter than 500 nm and only during the first 30 minutes of dark adaptation. Limitations of the PMT cathode radiant sensitivity, however, prevented accurate analysis in the range 700–720 nm. We recommend that future research be conducted using a PMT with a wider spectral range (i.e., the H7422P-50, 380–890 nm) to more accurately observe the full extent of the wound-induced UPE. Additionally, a selection of narrow, optical band pass filters would further serve to identify specific emitters within this spectral range.

In contrast, leaf wound-induced UPE was significantly suppressed when placed under anoxic stress (N_2_ gas) suggesting that wound-induced lipoxygenase reactions are inhibited under anoxic atmospheric conditions. The reduced UPE observed seems to corroborate recent experimental data [16,18] that has shown that inhibition of lipoxygenase production through use of chemical agents directly effects singlet chlorophyll and singlet oxygen luminescence. Determination of the exact sources of the UPE detected at wavelengths longer than 650 nm requires greater spectral resolution on the experimental setup. It was also observed that N_2_ incubation did not significantly reduce the dark adaptation fluorescence nor pre-wounding steady state basal photon counts.

Photon counting statistics of pre-wound (aerobic & anoxic) and anoxic wounding photon counts were found to closely approximate a Poisson distribution.

These observations contribute to the body of plant wound-induced luminescence research and provide a novel methodology to measure this phenomenon *in vivo* under both aerobic and anoxic conditions.

## Supporting information

S1 Fig *Spathiphyllum* single filter dark adaptation decay spectral analysis. Normalized, 10-point smoothed spectral analysis of dark-adapting *Spathiphyllum* leaves in air using various optical filters (see Table 1). Inset shows subtle decay observed in first 30 min after placing plant in the dark using SP500 filter.
(TIF)

S2 Fig Pre-wound filter wheel spectrum measurements (#2–#5) recorded every 15 minutes (900 s). Measurement start times: (A) 900 s, (B) 1800 s, (C) 2700 s and (D) 3600 s.
(TIF)

S3 Fig Pre-wound filter wheel spectrum measurements (#6–# 8) recorded every 15 minutes (900 s). Measurement start times: (A) t = 4,500 s, (B) t = 5,400 s, and (C) t = 6,300 s.
(TIF)

S4 Fig Box Plots of filter wheel spectral data. A. Pre wound spectral data. B. Post wound spectral data.
(TIF)

S5 Fig *Spathiphyllum* single filter aerobic wounding spectral analysis. Normalized, 10-point smoothed spectral analysis of *Spathiphyllum* aerobic wounding. Optical filters and associated wavelength as indicated and listed in Table 1.
(TIF)

S6 Fig Post-wound filter wheel spectrum measurements #2–#5. Measurements taken at: (A) ≈ 7,385 s, (B) t ≈ 7,505 s, and (C) t ≈ 7,625 s, and (D) final measurement at the end of testing (t = 4.375 hrs).
(TIF)

S7 Fig Images of leaf used for filter wheel wound spectrum measurements. (A) Leaf lower epidermis (wound side) showing previous healed wound, and new wound site. (B) Leaf upper epidermis (PMT facing side) showing old and new wounds. (C) High magnification CCD image capture of wound site. Circle is approximate image area (19.64 mm^2^).
(TIF)

S1 Table Filter wheel spectral analysis data. (TIFF)
