# Supplementary figures and images for: Leaf wound induced ultraweak photon emission is suppressed under anoxic stress: Observations of Spathiphyllum under aerobic and anaerobic conditions using novel in vivo methodology
Source: PLoS One. 2018 Jun 14;13(6):e0198962. doi: 10.1371/journal.pone.0198962 (PMC6002245; doi:10.1371/journal.pone.0198962)

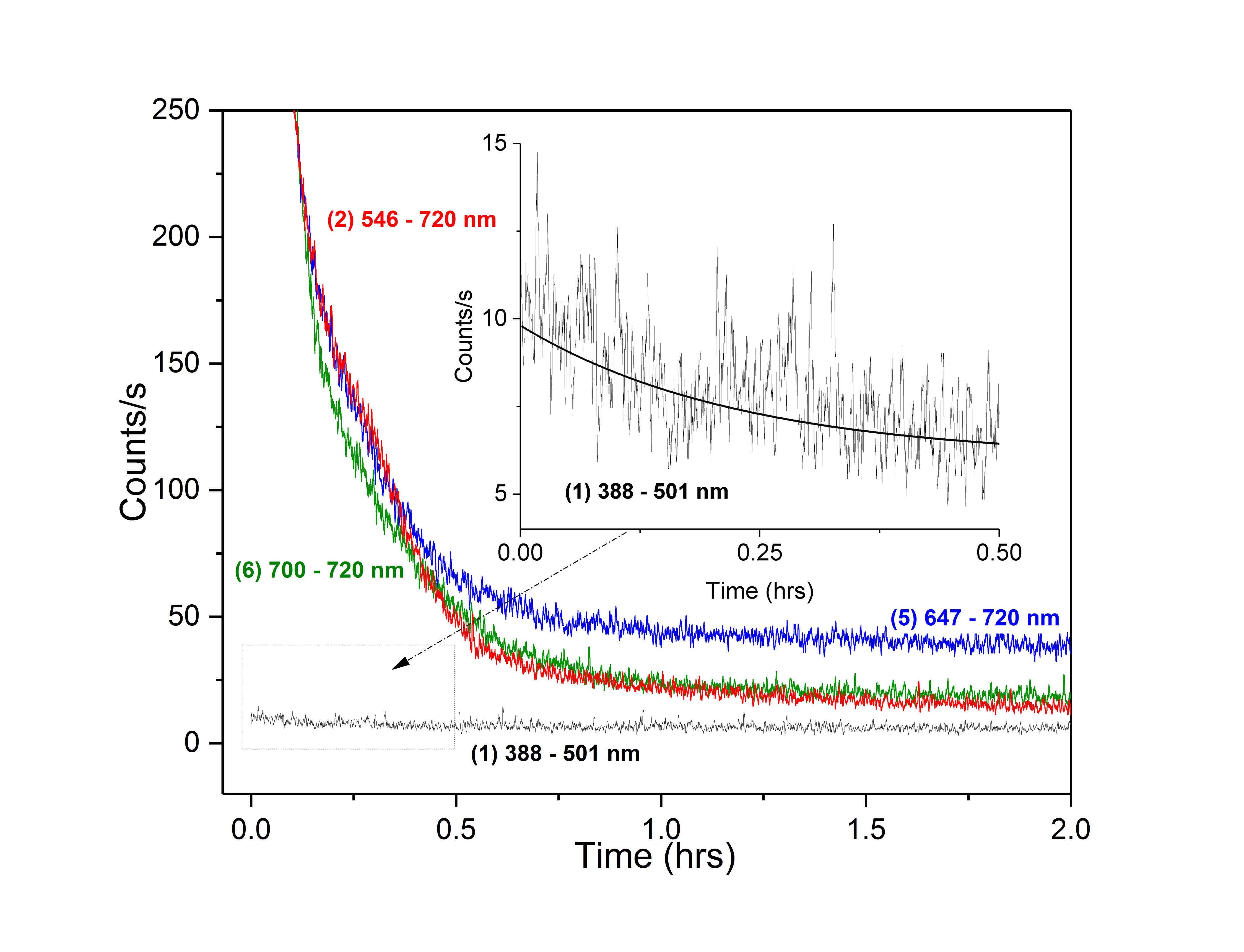

Supplement: S1 Fig — Normalized, 10-point smoothed spectral analysis of dark-adapting Spathiphyllum leaves in air using various optical filters (see Table 1). Inset shows subtle decay observed in first 30 min after placing plant in the dark using SP500 filter. (TIF) [file pone.0198962.s001.tif]

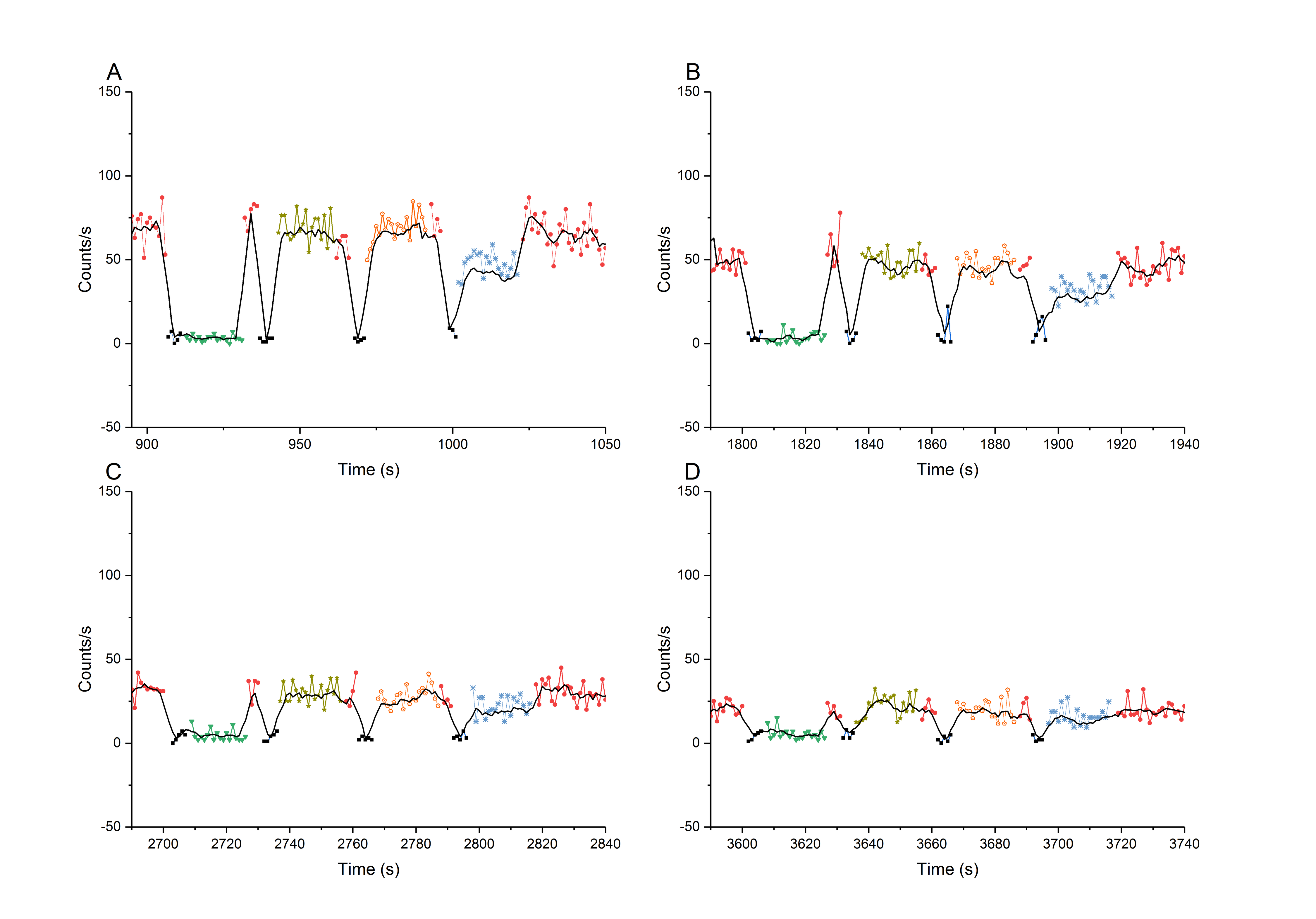

Supplement: S2 Fig — Measurement start times: (A) 900 s, (B) 1800 s, (C) 2700 s and (D) 3600 s. (TIF) [file pone.0198962.s002.tif]

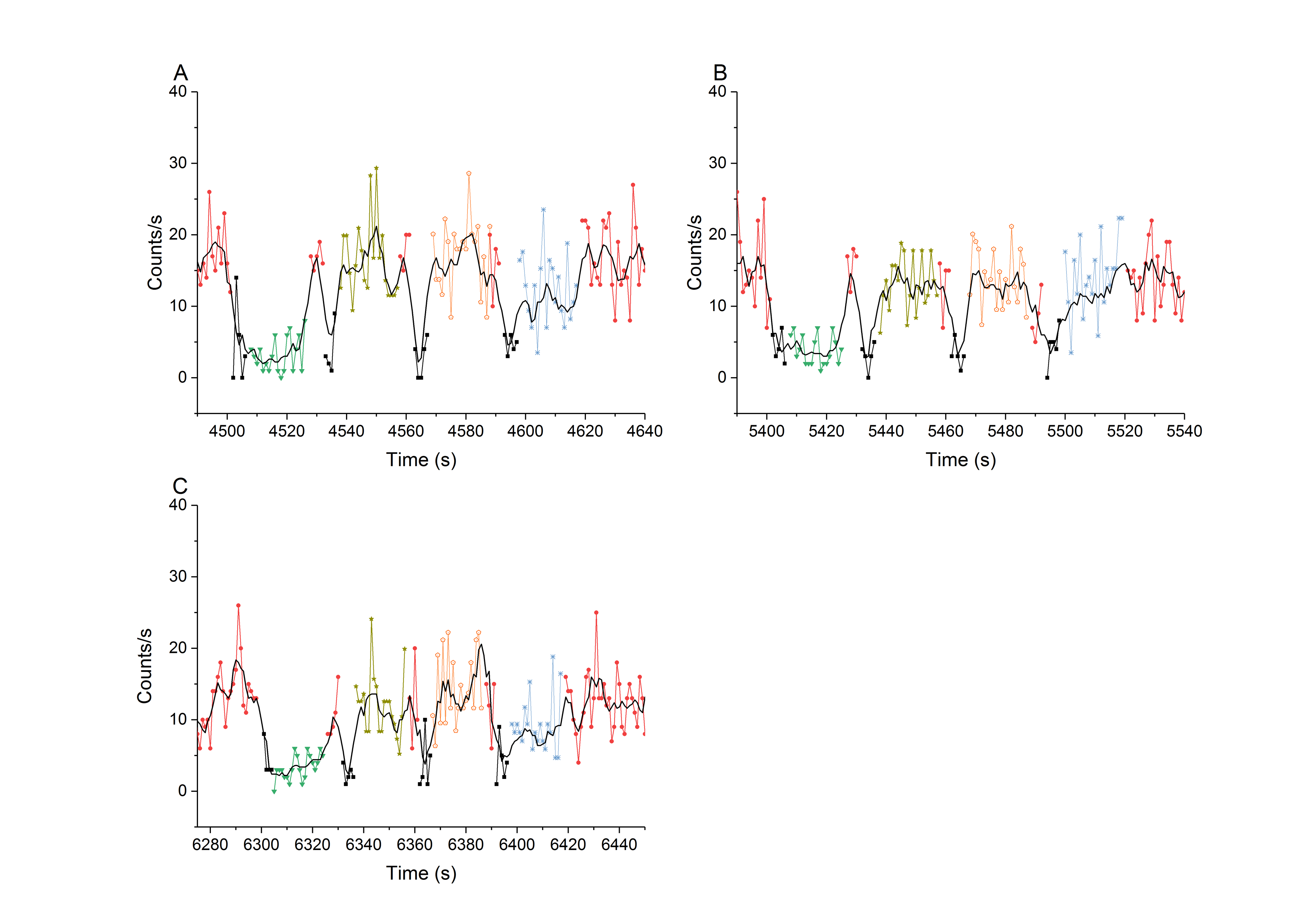

Supplement: S3 Fig — Measurement start times: (A) t = 4,500 s, (B) t = 5,400 s, and (C) t = 6,300 s. (TIF) [file pone.0198962.s003.tif]

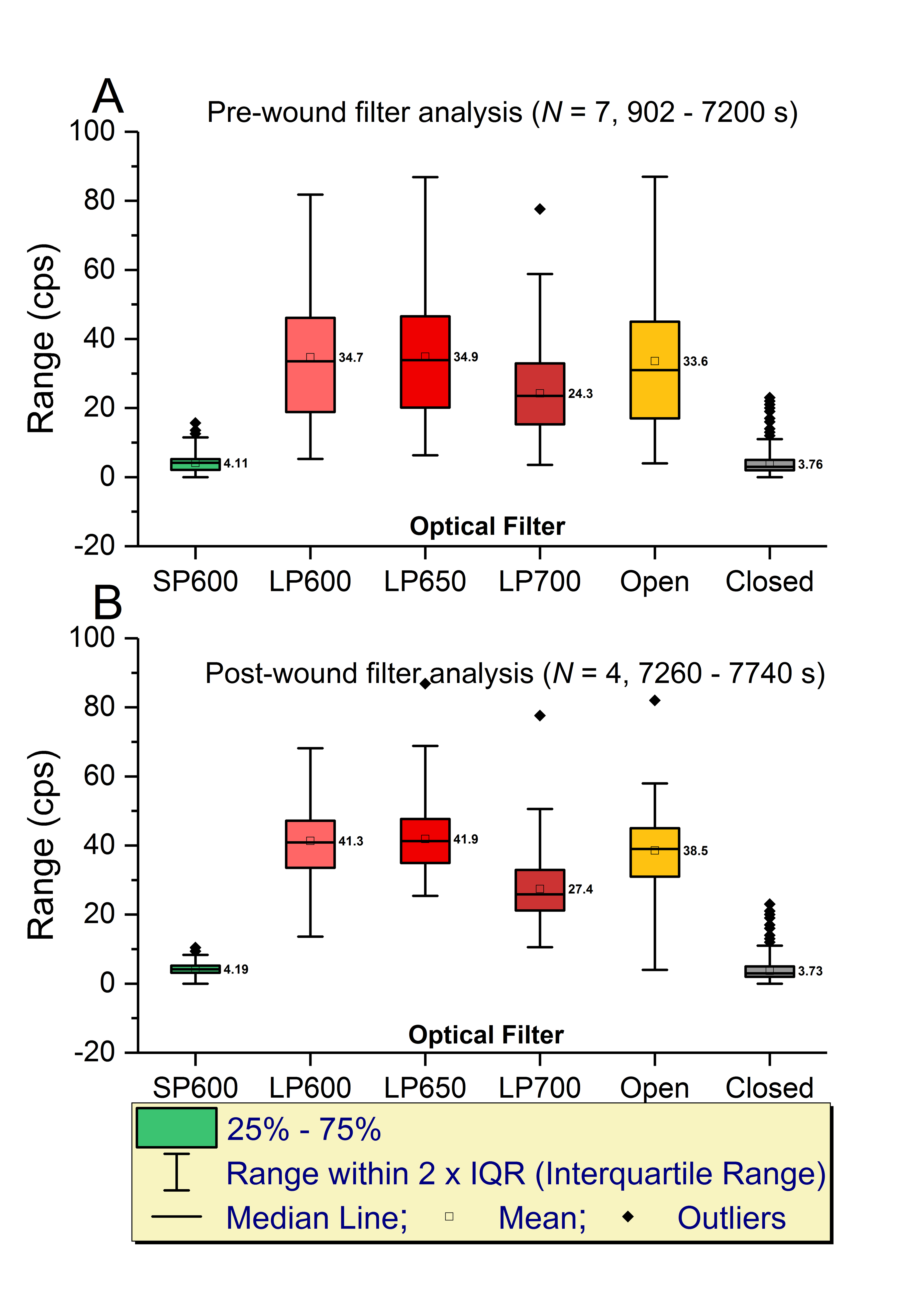

Supplement: S4 Fig — A. Pre wound spectral data. B. Post wound spectral data. (TIF) [file pone.0198962.s004.tif]

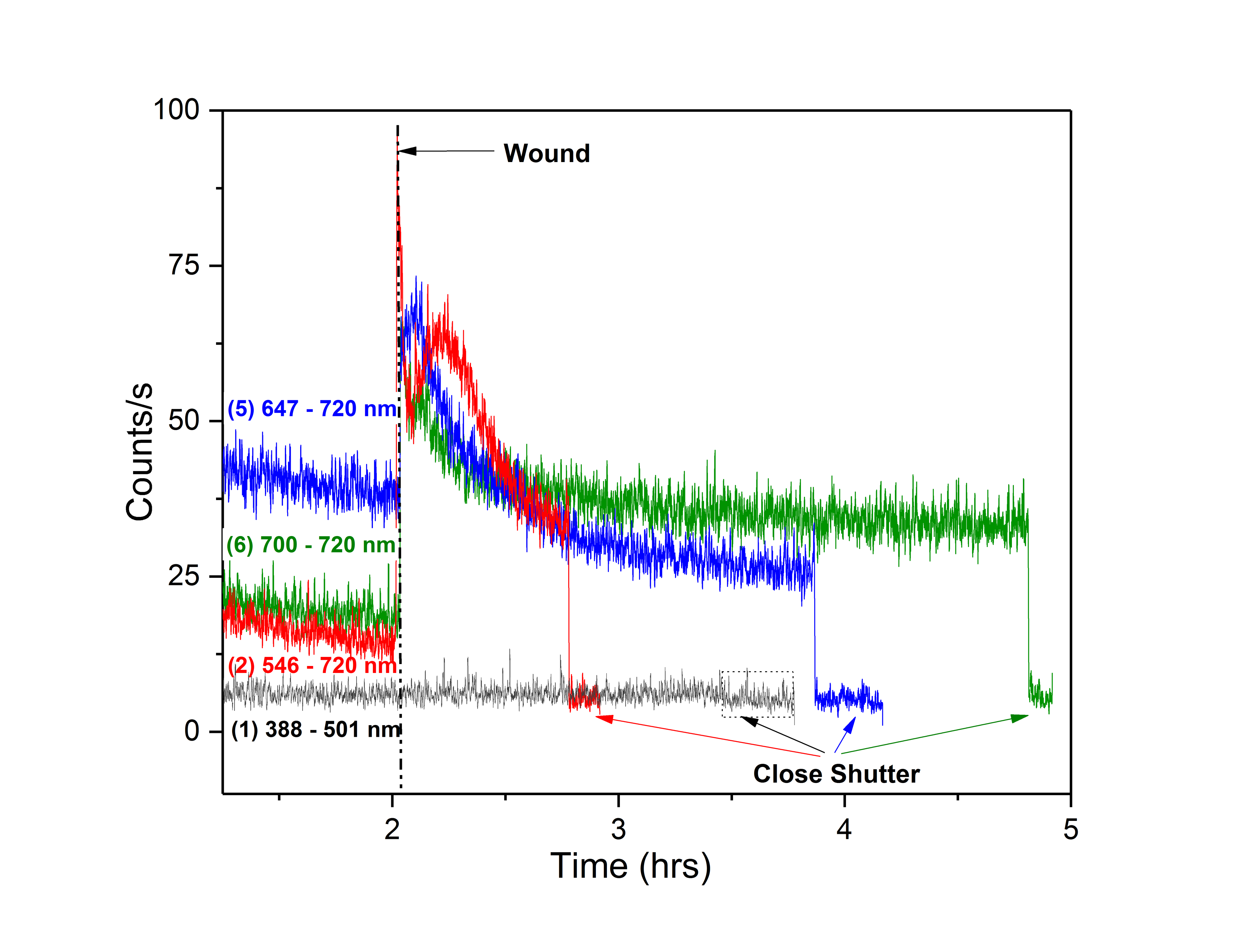

Supplement: S5 Fig — Normalized, 10-point smoothed spectral analysis of Spathiphyllum aerobic wounding. Optical filters and associated wavelength as indicated and listed in Table 1. (TIF) [file pone.0198962.s005.tif]

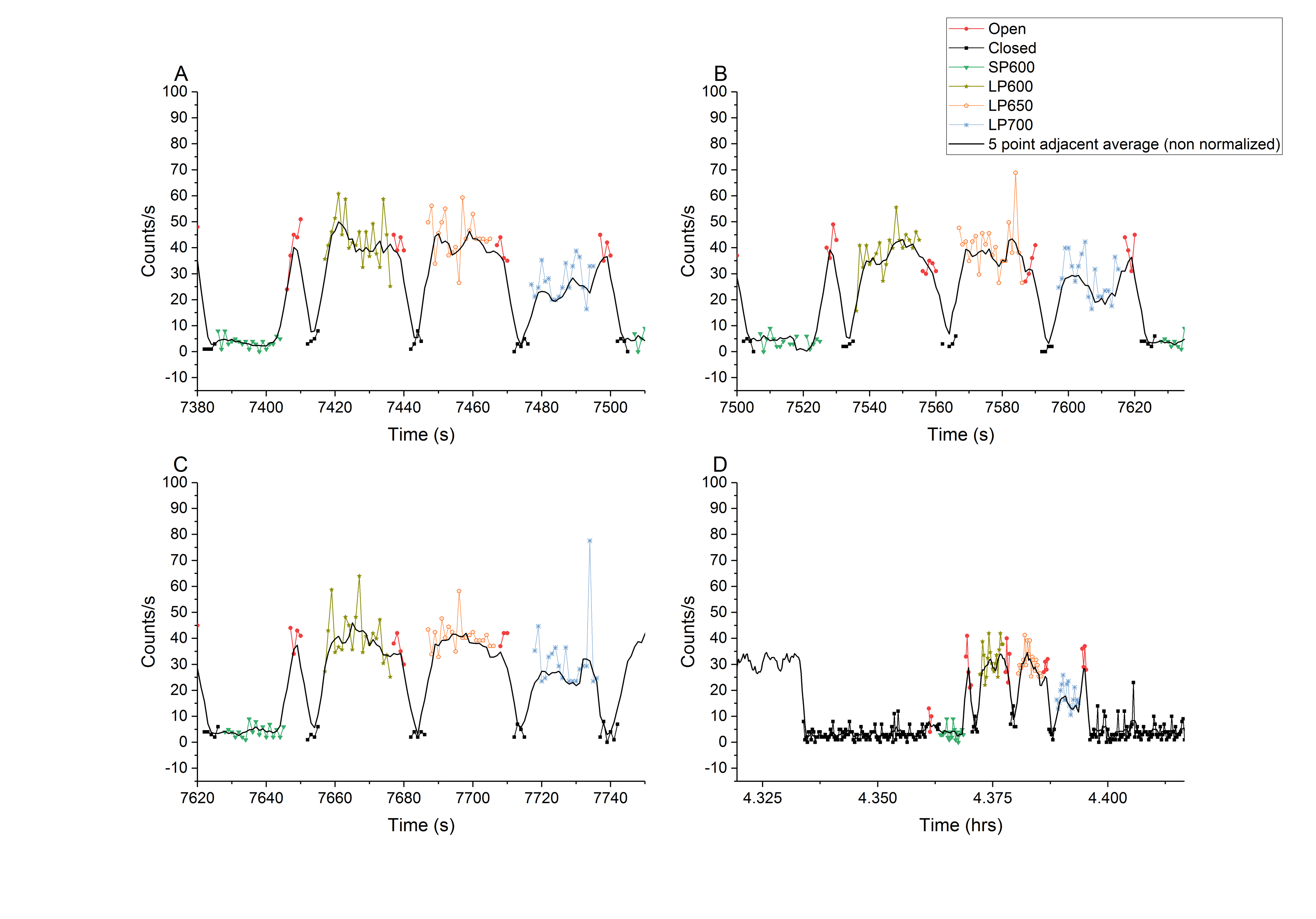

Supplement: S6 Fig — Measurements taken at: (A) ≈ 7,385 s, (B) t ≈ 7,505 s, and (C) t ≈ 7,625 s, and (D) final measurement at the end of testing (t = 4.375 hrs). (TIF) [file pone.0198962.s006.tif]

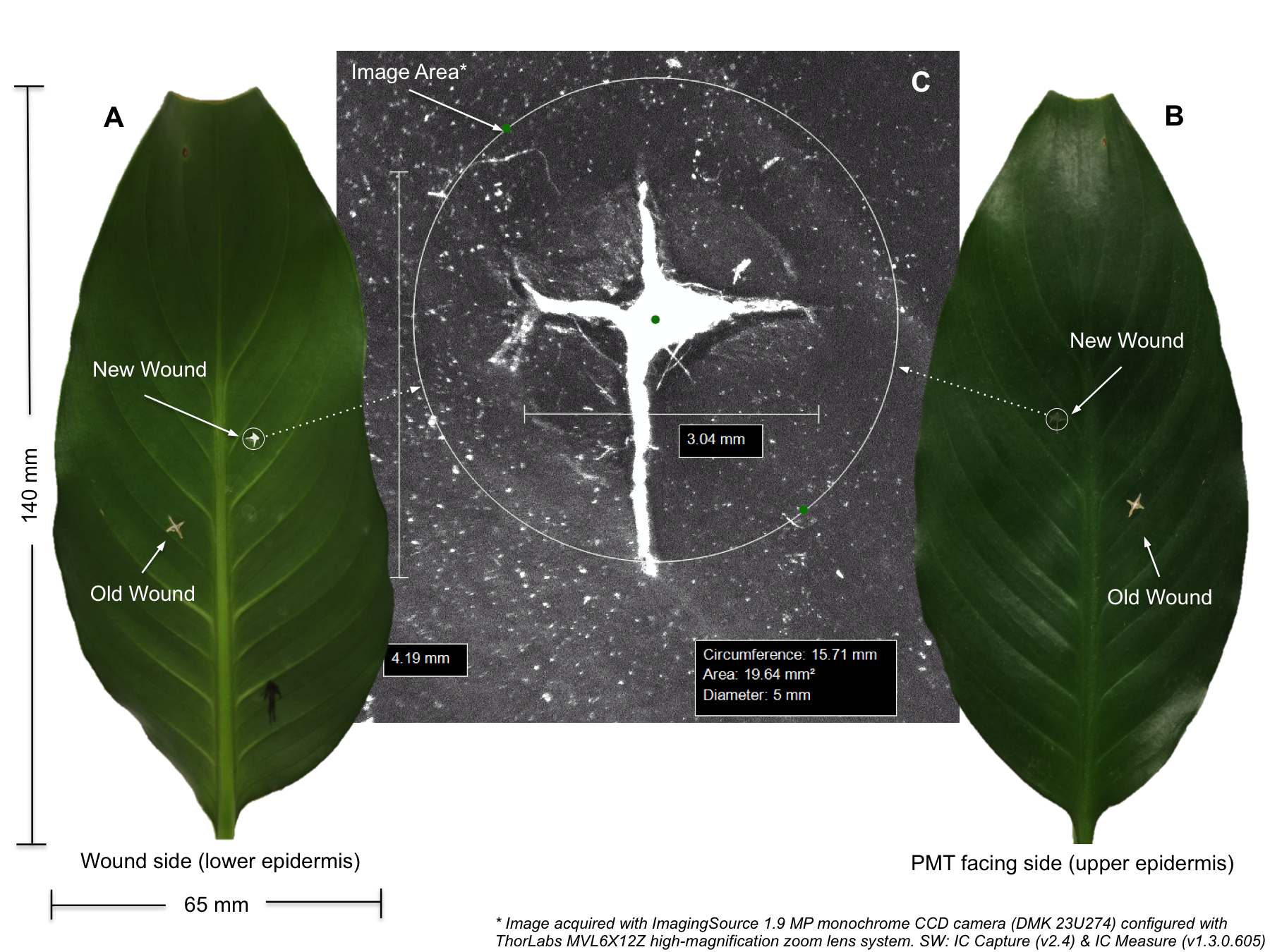

Supplement: S7 Fig — (A) Leaf lower epidermis (wound side) showing previous healed wound, and new wound site. (B) Leaf upper epidermis (PMT facing side) showing old and new wounds. (C) High magnification CCD image capture of wound site. Circle is approximate image area (19.64 mm2). (TIF) [file pone.0198962.s007.tif]

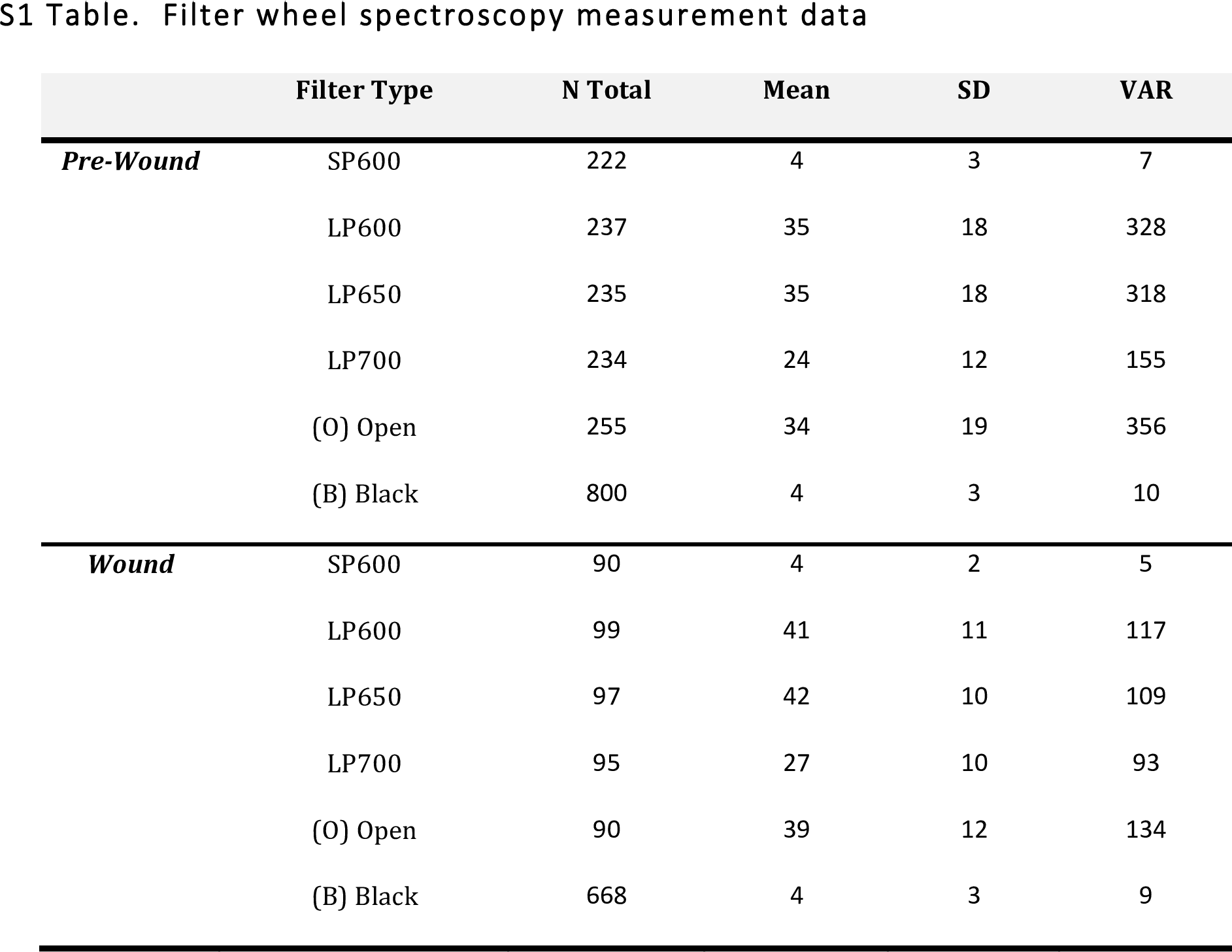

Supplement: S1 Table — (TIFF) [file pone.0198962.s008.tiff]
